# Effects of Antibiotic Use on Saliva Antibody Content and Oral Microbiota in Sprague Dawley Rats

**DOI:** 10.3389/fcimb.2022.721691

**Published:** 2022-01-31

**Authors:** Xi Cheng, Fuming He, Misi Si, Ping Sun, Qianming Chen

**Affiliations:** ^1^ Stomatology Hospital, School of Stomatology, Zhejiang University School of Medicine, Clinical Research Center for Oral Diseases of Zhejiang Province, Key Laboratory of Oral Biomedical Research of Zhejiang Province, Cancer Center of Zhejiang University, Hangzhou, China; ^2^ Department of Stomatology, People’s Hospital of Leshan, Leshan, China

**Keywords:** oral microbiome, diversity, high-throughput 16S rRNA sequencing, function prediction, antibiotic

## Abstract

Antibiotics are often used to treat systemic diseases not associated with the oral cavity. This application of antibiotics may affect the healthy oral microbiota community, as it destroys the balance between specific bacterial populations throughout the ecosystem and may lead to dysbacteriosis. We hypothesized that the effects on antibiotics on oral microbiota regulation and function would affect antibody content in saliva, depending on the antibiotic type. To address this, a total of 24 Sprague Dawley rats (divided into 4 cages, 6 per pen) were administered amoxicillin (AMX), spiramycin (SP), metronidazole (MTZ), or water (control) daily for 14 days (gavage). After treatment was completed, high-throughput sequencing of 16S rRNA genes was used to determine changes in the composition, metabolic function, and diversity of oral microbiota in the rats. Enzyme-linked immunosorbent assay was used to detect antibodies in saliva, including SIgA, IgG, and IgM. Results showed that AMX, MTZ, and SP significantly affected oral microbiota composition, diversity, and metabolic function in rats. AMX induced substantial changes in the rat salivary antibody concentrations. At the genus level, the relative abundance of *Rothia* and *Haemophilus* was higher in the AMX group than in the other groups. In conclusion, antibiotics-induced changes in oral microbiota populations may be associated with changes in salivary antibody concentrations. However, the specific interaction mechanisms remain unknown, and it is still unclear whether significant changes in the oral microbiota cause changes in salivary antibody concentrations or *vice versa*.

## Introduction

The oral microbiota is one of the human body’s five primary microbial reservoirs (intestine, oral cavity, skin, nasal cavity, and urogenital tract) (Gao et al., 2018). Oral disorders like as caries, periodontal disease, Pericoronitis, and jaw osteomyelitis are caused by an imbalance of the oral microbiota, which is also linked to cancers, diabetes, rheumatoid arthritis, and cardiovascular disease, all of which have a substantial influence on human health (Chattopadhyay et al., 2019). Some doctors often treat bacterial infections with antibiotics regardless of the site of infection, which will inevitably affect the intricate and delicate microbiota ecosystem colonizing the oral cavity (Dar-Odeh et al., 2010). Previous studies have examined the effects of various antibiotics on gut microbiota (Takesue et al., 2002), yet few have focused on oral microbiota. To examine the effect of antibiotics on the oral microbiota, we treated Sprague Dawley rats with three antibiotics, namely amoxicillin (AMX), metronidazole (MTZ), and spiramycin (SP), *via* gavage. These antibiotics were chosen because they represent different classes and are commonly used to treat human infections and other bacterial targets (**Tables 1**, **S2**). High-throughput 16S rRNA genes sequencing was used to determine changes in oral microbiota composition, metabolic function, and diversity in the oral cavity of the rats at 14 days after gavage.

**Table 1:** Diversity forms.

Sample\Estimators	shannon	simpson	ace	chao	coverage
a5	2.634229	0.129787	350.9616	349.3947	0.998136
m1	2.510799	0.126589	387.4882	357.7333	0.997612
m3	1.849725	0.271049	367.5412	231.6667	0.998866
c5	2.166963	0.170261	364.8633	274.6	0.998263
s1	1.881613	0.387539	334.6149	394.0909	0.997879
c1	3.318317	0.074409	367.1817	364.122	0.998485
c4	2.493486	0.120342	348.3256	239.9643	0.998446
s5	3.627431	0.095353	710.6693	721.2877	0.997062
s6	2.799704	0.09325	571.2998	447.717	0.997057
s2	2.644053	0.112912	418.201	368.6207	0.997031
s3	2.754805	0.152288	257.5413	276.5	0.998755
m6	1.971378	0.232935	233.2815	181.3684	0.998881
c2	2.626869	0.128288	369.2054	349.4375	0.997836
a4	2.510635	0.14488	399.253	332.5714	0.99777
c3	2.512929	0.156833	379.9679	304.7857	0.997826
a1	2.437181	0.144455	339.8593	337.2857	0.997789
s4	2.596664	0.12129	677.2424	473.9574	0.996295
m4	1.857436	0.284274	336.0108	212.9565	0.998201
a6	1.862487	0.205854	447.9996	289.375	0.997969
c6	2.343833	0.13477	265.6663	230.8889	0.99861
a3	2.920933	0.103666	426.2607	406.6438	0.997933
m2	2.492253	0.129232	469.9545	339.375	0.997641
m5	2.169715	0.197322	369.8203	278	0.998138
a2	2.914677	0.088877	382.5884	366.4884	0.997536

Saliva antibody concentration is another indicator of oral health in humans (Defabianis and Re, 2003). Increased antibody content in saliva enhances immunity in the organism. However, it also leads to damage to the oral cavity, such as progression of periodontal disease and gingivitis (Mikuls et al., 2009). Secretory immunoglobulin A (SIgA) is considered as the predominating antibody in saliva. It is the body’s first line of defense against bacteria and can fight foreign bacterial invasion (Wang et al., 2020). Immunoglobulin G (IgG) is the most abundant immunoglobulin in human serum and extracellular fluid, accounting for approximately 75% to 80% of plasma total immunoglobulins (Tsilibary et al., 2018). Finally, IgM is beneficial for antigen agglutination (Huang et al., 2017). Enzyme-linked immunosorbent assay (ELISA) uses the characteristics of specific binding between antigen and antibody to detect samples; it can visualize the presence of a specific antigen or antibody and can be quantitatively analyzed by the color depth, making it suitable for the study of saliva antibody content (Konstantinou, 2017). Literature search revealed that there are only few studies on the effect of different types of antibiotics on salivary antibody concentrations in organisms, and that SIgA, IgG, and IgM are the most representative saliva antibodies; therefore, we used the ELISA technique to explore the changes in rat salivary antibody concentrations after the administration of different types of antibiotics.

In addition, our overall goal was to evaluate the effects of different types of antibiotics on oral micro-ecology. Therefore, we also investigated the association between changes in antibody concentration and changes in the abundance of certain strains of oral microbiota. We found changes in the relative abundance of *Rothia* and *Haemophilus* as well as salivary antibody concentrations in the saliva of rats in the AMX group. However, the increased abundance of *Acinetobacter, Staphylococcus*, and *Lactobacillus* in the SP group did not lead to changes in antibody concentrations. Thus, further exploration of the correlation between the two indicators is needed.

## Material and Methods

### Ethics Statement

Animal experiments were conducted at the Central Laboratory of Leshan People’s Hospital (Leshan, Sichuan Province, China) and approved by the ethics committee of Leshan City People’s Hospital (authorization number 2018-11-03-00089). All experiments were performed according to the approved guidelines and regulations for the care and use of animals.

### Animals and Housing

Briefly, 8-week-old male specific-pathogen-free Sprague Dawley rats (n=24) were purchased from Dashuo Company (Chengdu, Sichuan, China). The rats were kept under controlled environmental conditions with 12-h light/dark cycle, temperature of 20.5 ± 0.3°C, and relative humidity of 51.3 ± 3.1%. The animals were provided free access to water and feed throughout the experiment (Dashu Company, Chengdu, Sichuan, China). Animal weight, feed intake, and water intake were monitored throughout the study.

### Experimental Design and Sample Collection

**Figure 1:**
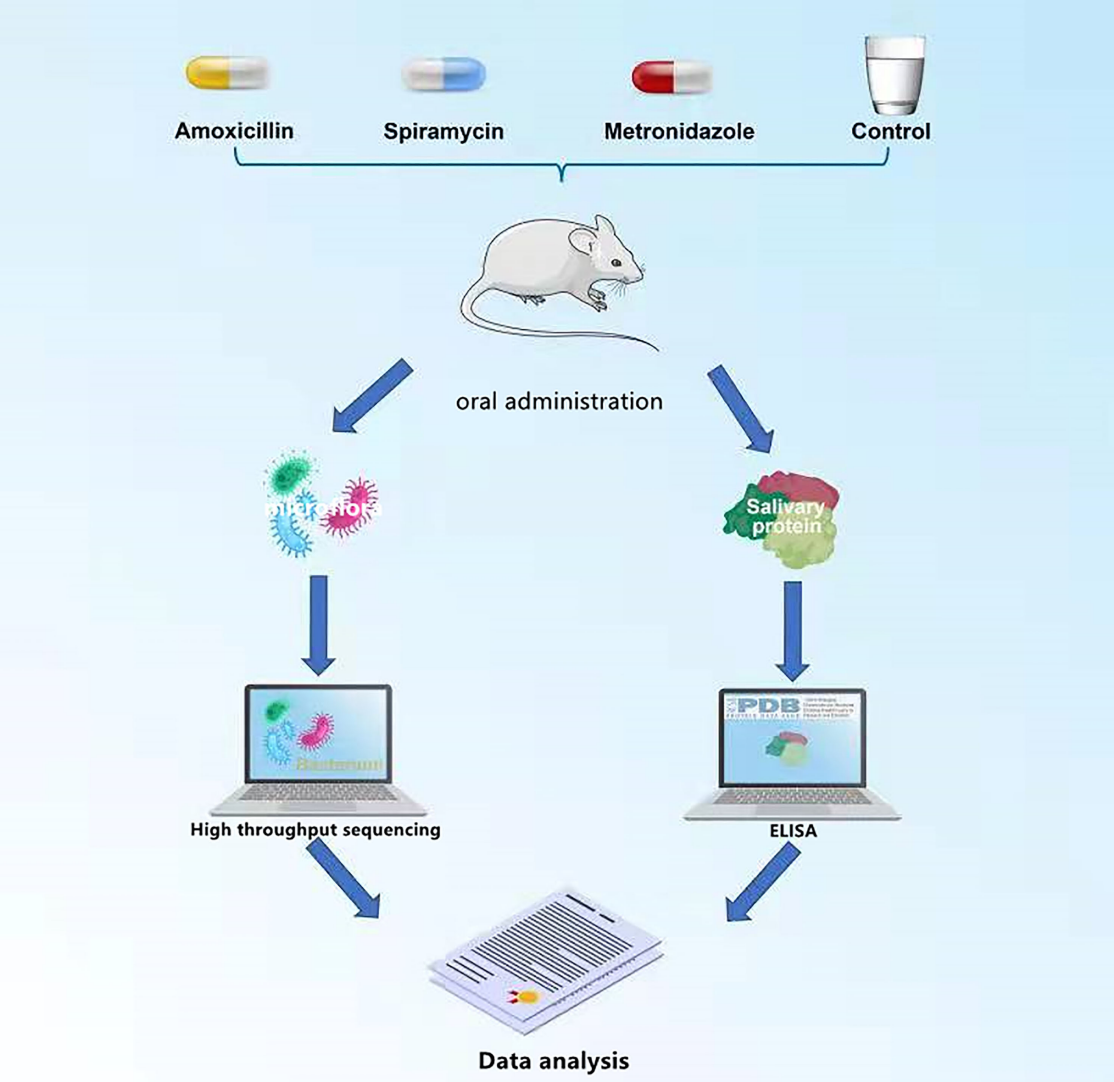
Research mind map.

The rats were weighed and randomly divided into four groups: AMX, MTZ, SP, and control groups (six rats in one cage per group), according to the dispensing in the Central Laboratory of Peoples Hospital of Leshan (Cheng et al., 2018). The rats received a daily dose of SP 0.25 g/kg/day, MTZ 0.19 g/kg/day, AMX 0.20 g/kg/day, or purified water by oral gavage for 14 days (14 days was the maximum period for routine use of antibiotics). After the treatment period, the rats were anesthetized, and oral microbiota samples were collected using a medical cotton ball. Saliva samples were collected using a micropipette (the rats were tied upside down on an operating table, and food was used to induce saliva secretion; 50 mM saliva was collected from each rat). All samples were collected and kept under sterile conditions on -80°C dry ice until they were tested (Cheng et al., 2018). The whole experimental process is shown in **Figure 1**.

### ELISA Test

Rat SIgA, IgM, and IgG ELISA kits (Tengfei company, Shenzhen, China) were used to measure the concentrations of SIgA, IgM, and IgG in samples according to the manufacturer’s protocol. Saliva samples from the four groups were cultured in four 24-well culture plates (Tengfei Incorporated, Shenzhen, China). The media in the second, third, and fourth cleaned culture plates were replaced with IgG (0, 2.5, 5, and 10 mM), rat SIgA (0, 2.5, 5, and 10 mM), and rat IgM (0, 2.5, 5, and 10 mM), respectively. The sample was then incubated in 5% CO2 at 37°C for 24 h. A microplate spectrometer (Tengfei Company, Shenzhen, China) was used at a wavelength of 450 nm to determine the OD values. The final concentration of the supernatant was calculated based on the standard curve.

### DNA Extraction,PCR Amplification and Illumina MiSeq Sequencing

E.Z.N.A was used to extract microbiota DNA from the rat samples. Soil DNA Kit (OMEGA bio TEK, Norcross, GA, USA) was used according to the manufacturer’s protocols. The final DNA concentration and purity were determined by a NanoDrop 2000 UV-vis spectrophotometer (Thermo Scientific, Wilmington, USA). DNA quality was evaluated *via* 1% agarose gel electrophoresis. The V4-V5 hypervariable regions of the bacterial 16S rRNA gene were amplified with primers 338F (5′-ACTCCTACGGGAGGCAGCAG-3′) and 806R (5′-GGACTACHVGGGTWTCTAAT-3′) using a thermocycler PCR system (GeneAmp 9700; ABI, USA). PCR amplifications were conducted using the following program: 3 min of denaturation at 95°C, followed by 27 cycles of 55°C annealing for 30 s, 72°C extension for 45 s, and 72°C extension for 10 min. The reaction volume was a triplicate 20 µL mixture containing 4 µL of 5xFastPfu Buffer, 2 µL of 2.5 mM dNTPs, 0.8 µL of each primer (5 µM), 0.4 µL FastPfu Polymerase, and 10 ng template DNA. The PCR product was extracted from 2% agarose gel and purified using an AXYPREP DNA Gel Extraction Kit (AXYGEN BioScIsStices, Los Angeles City, USA) and quantified by using quantitative Floor.-ST (PROMEGA, USA), according to the manufacturer’s protocol.Amplifiers information were collected on an Illumina MiSeq platform (Illumina, San Diego, USA). Majorbio Bio-Pharm Technology Co., Ltd. supplied the standard. (Shanghai, China). The original reads were stored in the NCBI sequence reading archive (SRA) database (accession number: PRJNA533200).

### Sequence Analysis

Majorbio I-Sanger Cloud Platform, a free online platform, was used to evaluate the data. (www.i-sanger.com). Raw fast files were demultiplexed, quality-filtered by Trimmomatic, and merged by FLASH using the following criteria: I reads truncated at any site received an average quality score of 20 across a sliding window of 50 bp, (ii) primers were carefully matched to enable two nucleotide mismatching, and (iii) merging sequences had overlapping lengths greater than 10 bp based on overlapping sequences. UPARSE (version7.1, http://drive5.com/uparse/) was used to perform operational taxonomic unit (OTU) clustering using a 97 percent similarity cut-off (Schloss and Westcott, 2011). Chimeric sequence was analyzed by RDP classifier algorithm (http://rdp.cme.msu.edu/) against the Silva (SSU123) 16S rRNA gene (Bacci et al., 2015). R software version 3.6.2 was used to plot the light curves and heat maps. Chao 1 and ACE indices were measured using the Mothur software (Gihring et al., 2012). Beta diversity was assessed by principal coordinate analysis and multivariate analysis of variance based on the Bray-Curtis distance (PERMANOVA) (Anderson, 2014). The PICRUSt algorithm was used to estimate probable functions of the rat oral microbiome, which was then compared to the Kyoto Encyclopedia of Genes and Genomes (KEGG) database (Kanehisa and Goto, 2000). The Wilcoxon test was used for statistical analysis, with the significance set at P < 0.05.

## Results

### Elisa Test Result

**Figure 2:**
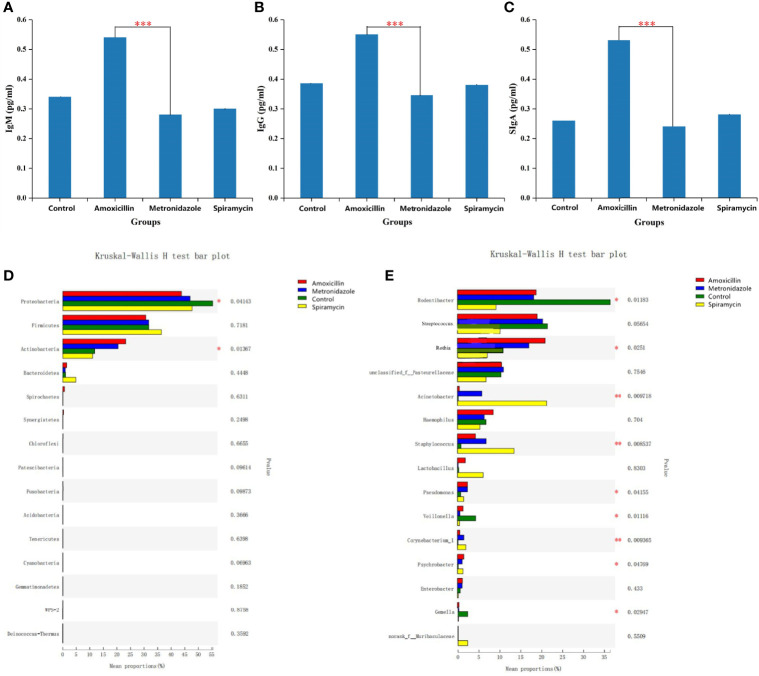
Statistical comparison of the ELISA experimental results **(A–C)** and relative abundance **(D, E)** of rats oral microbiota among the four groups. **(A–C)** SIgA,IgM,IgG content in all samples. **(D)** Comparison of dominant phyla in all groups. **(E)** Comparison of dominant genera in all the groups. Differences were considered statistically significant at *p<0.05; **p<0.01; ***p<0.001 level.

To examine the effect of antibiotics on antibody content in rat saliva, ELISA was performed to determine the mean levels of IgG, IgM, and SIgA in all samples. As shown in **Figures 2A**, respectively, IgG, IgM, and SIgA were detected in all samples. Comparisons between duplicate measurements of each immunoglobulin showed significant differences between these values. The mean IgG, IgM, and SIgA levels were higher in the AMX group than in the other groups (P < 0.05). Immunoglobulin levels in the other three groups were not constant (**Figures 2A–C**).

### Sequencing Output

In this study, high-throughput sequencing was used to characterize the microbiota composition and community structure of rat oral microbiota. An average of 18,570 high-quality reads were obtained in 24 samples after interference reduction. The reads, coverage, OTUs, and species richness of each example with a genetic distance of 3% are presented in Additional Materials **Tables S1**, **S2** and **Figures S1**–**S3**. R software was clustered at a 97% phylotypic similarity level, and rarity, Shannon-Wiener, and rank abundance curves were generated.

#### α Diversity of Oral Microflora

**Figure 3:**
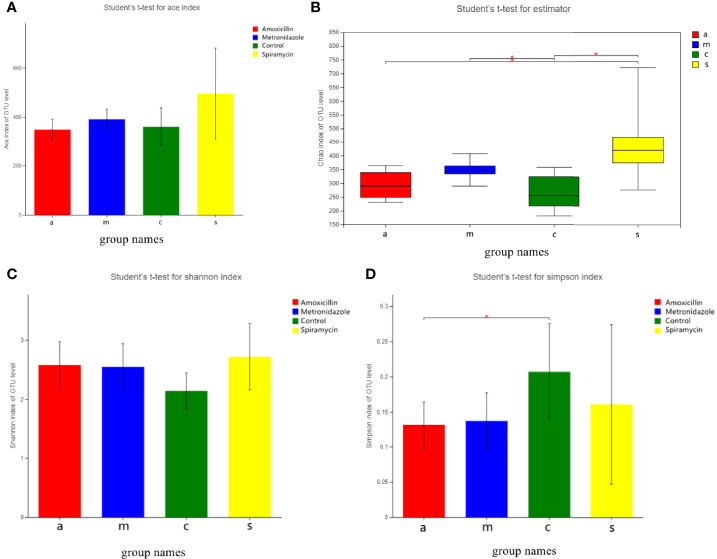
**(A)** Bacterial community richness (measured by ACE index) in all the groups. **(B)** Bacterial community richness (measured by Chao index) in all groups. **(C, D)** Bacterial community diversity (measured by Shannon, Simpson index) in all the groups. Differences were considered statistically significant at *p < 0.05; **p < 0.01 level.

**Figure 3** shows the alpha diversity of oral microbiota in the four groups of rats administered different types of antibiotics. AMX and MTZ increased the Chao(Chao1 estimator) and ACE(ACE estimator) indices of bacteria in each group (**Figure 3A**), indicating that these antibiotics enhanced the abundance of oral microbiota communities in rats. Shannon and Simpson indices were also consistent with this finding (**Figure 3C**) that compared with that in the control group, microbiota communities in rat oral cavity were increased in the AMX, MTZ, and SP groups.

### Taxonomic Composition

**Figure 4:**
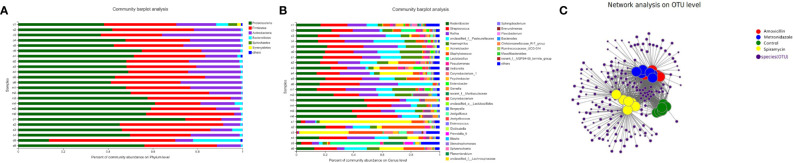
Oral bacterial community at the phylum **(A)** and genus **(B)** level. (Phyla or genera with abundance of less than 1% abundance were merged into ‘others’. **(C)** Network-based analysis of oral microbial communities in Amoxicillin, Metronidazole, Control, Spiramycin groups.

According to 16S rRNA gene sequences, 28 prokaryotic clades were identified (**Figure 4**). In the MTZ group, *Proteobacteria* was predominant phylum, constituting 47.96–56.96% of the 16S rRNA gene sequences. The second-most abundant phylum was *Firmicutes*, which represented 37.15–20.42% of the samples.

In the group treated with AMX, the most abundant phyla were *Proteobacteria* (51.31–41.27%), *Firmicutes* (40.31–31.25%), and *Bacteroidetes* (27.13–15.61%); moreover, *Firmicutes* and *Actinobacteria* were predominant, accounting for 64.41–43.1% of each sample. At the genus level, there were a total of 496 different bacterial genera in the detected OTUs (**Figure 4**). In the AMX group, 350 genera were detected, whereas in the MTZ group, 372 genera were detected. In contrast, a more diverse group of 384 genera was observed in the SP group. At the phylum level, only *Proteobacteria* showed a significant difference in relative abundance in rat oral microbiota between the three groups (**Figure 4**). However, at the genus level, the relative abundance of *Rodentibacter, Streptococcus, Rothia, Acinetobacter, Staphylococcus*, and *Lactobacillus* was significantly different between the four groups (**Figure 2**). These findings are consistent with those of previous studies of rat gut microbiota (Behr et al., 2017). Different types of antibiotics cause different structural characteristics of the gut microbiota. Thus, the host bacteria are classified into three categories depending on the target microbiota of these antibiotics. (See supplementary material **Figure S5** for stacked column plots comparing the relative abundance of microbiome taxa across all groups at the class, order, family, and species levels.). All the sequences were assigned to a total of 1054 OTUs. OTU 236, OTU 225, OTU 502, and OTU 237 were the top four most abundant OTUs in all samples (See Additional Materials, **Figure S4**).

### Community Structures

**Figure 5:**
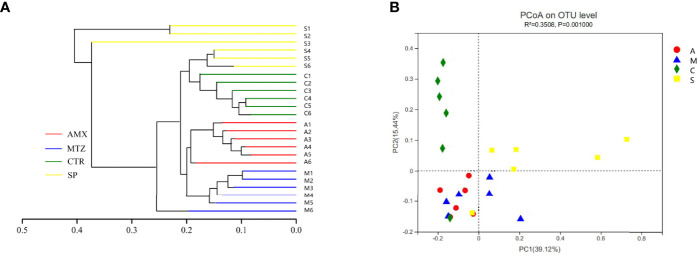
Relationship of the rats oral microbiota from different groups. **(A)** Clustering tree of the oral microbiotas in different rats from Amoxicillin (AMX), Metronidazole (MTZ), Control (Con), spiramycin (SP).Oral microbiota trees were generated using the UPGMA (unweighted pair group method with arithmetic mean)algorithm based on the Bray-Curtis distances generated by Mothur. **(B)** Principal coordinate analysis (PcoA) of community structure of the oral microbiotas of the four groups. Amoxicillin (red), Metronidazole (blue), Spiramycin (yellow) and Control (green) shapes represent the oral microbiotas from each groups, respectively. Distances between symbols on the ordination plot reflect relative dissimilarities in community structures.

Next, we examined the oral microbial communities of the 24 rat samples. The four groups were represented using Bray-Curtis distances in a dendrogram (**Figure 5**). Each branch on the tree represents a rat oral microbiota sample. The results showed that the clustering and phylogeny of the oral microbiota of rats differed sharply with the different types of antibiotics administered to each group. There was some crossover between the control and SP groups in the dendrogram. In addition, we studied the structure of the oral microbiota in four groups of rats using beta diversity analysis. In the typological analysis, each symbol represents one oral microbiota community (**Figure 5**). Similar to the results of clustering analysis, microbiota communities in the AMX and MTZ groups were clustered closely and separated from those in the SP and control groups. Principal axis 1 (PC1) explained the most considerable variation (39.12%). Next, we used a network-based analysis map to display the effects of the different types of antibiotics on the composition and structure of oral microbiota communities in rats (**Figures 4**, **S6**). The results were consistent with the dendrogram and beta diversity analysis results. These findings suggest that the administration of different types of antibiotics affects microbiota composition in rat oral cavity, but the oral microbiota structure of rats receiving SP was similar to that of the control group.

### Differences Gene Function in All Rats Oral Microbiota Samples

**Figure 6:**
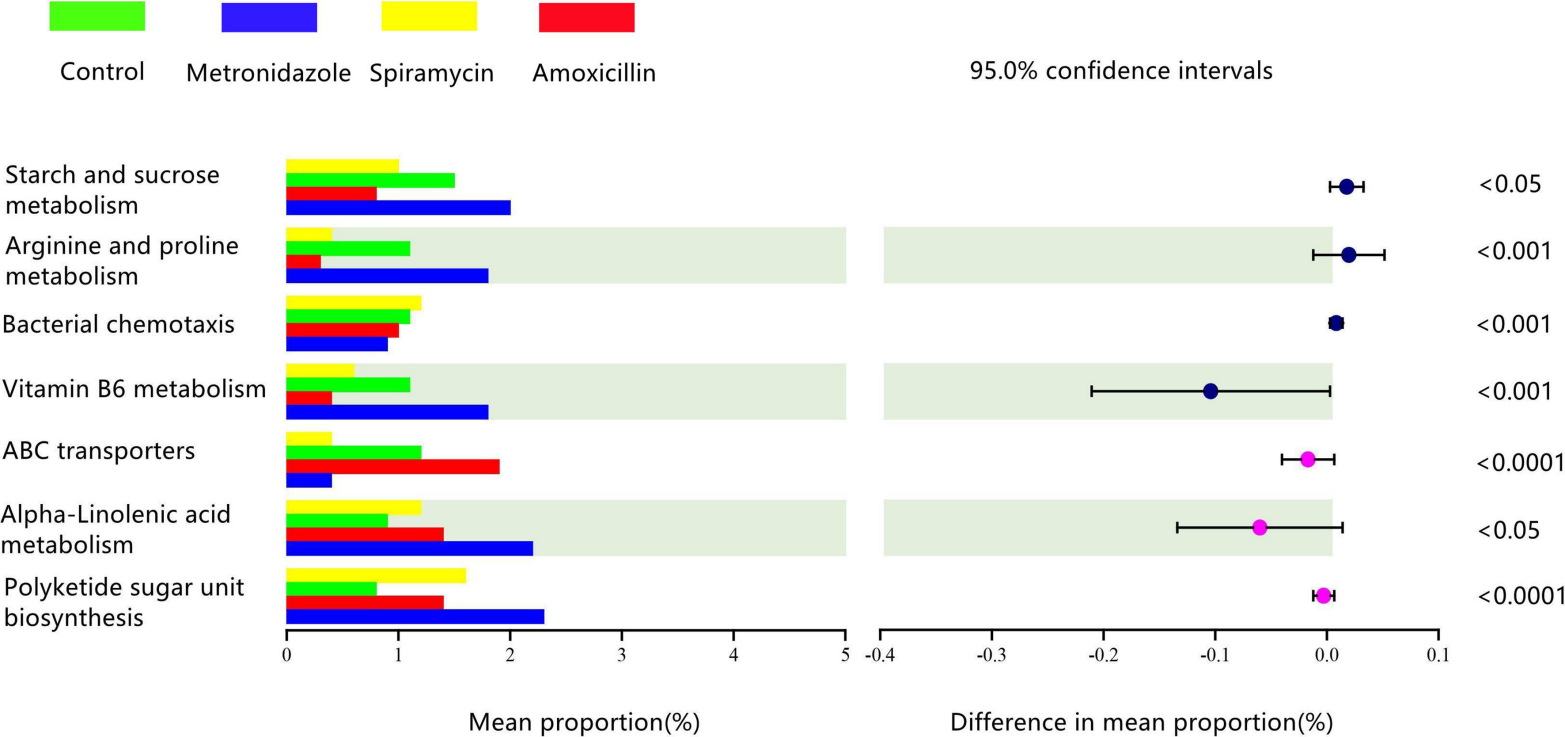
Comparison of predicted gene functions of rats oral microbiota between the Amoxicillin (AMX), Metronidazole (MTZ), Control (Con), spiramycin (SP) groups.

We used the PICRUSt algorithm to analyze the effects of different classes of antibiotics on oral microbiota function. We found that rat oral microbiota samples from the AMX, MTZ, control, and SP groups showed different KEGG profiles. α-Linolenic acid metabolism, polyketide sugar unit biosynthesis, vitamin B6 metabolism, arginine proline metabolism, and starch and sucrose metabolism were up-regulated in the MTZ group. In contrast, gene functional pathways involved in vitamin B6 metabolism, ABC transporters, and arginine and proline metabolism were reduced in the SP group (**Figure 6**).

## Discussion

As expected, the administration of AMX, MTZ, and SP for 14 days resulted in changes in the oral microbiota diversity of rats (**Figure 3**). However, the changes in the microbiota diversity were smaller following treatment with SP than those with the other antibiotics, which may indicate that macrolide antibiotics have a more significant effect on microbiota than β-lactam antibiotics. Antibiotic therapy is often considered to diminish the quantity and variety of bacteria, but our findings revealed the reverse. Antibiotic administration, regardless of the kind of antibiotic used, increased bacterial diversity, which is likely attributable to the formation of communities of antibiotic-resistant bacteria in organisms. This phenomenon has been noticed in previous research (Dudek-Wicher et al., 2018). An increase in oral microbiota diversity is not favorable to the host because the ecology of microbiota and functional pathways is closely related to primitive oral microbiota, taxonomic, and functional alpha diversity across microbiota communities is significantly correlated (Freire et al., 2021). Furthermore, increasing microbiota diversity in the oral cavity might result in more germs entering the air of the dental treatment room through the aerosol created by dental therapy, which has an influence on medical personnel and patients (Zemouri et al., 2020). Furthermore, the oral microbiota increases the risk of infection in routine dental surgery, such as periodontal surgery, root canal therapy, and tooth extraction. Most importantly, as the entrance to the digestive system, the oral cavity serves as a transportation hub connecting the inside and outside of the human body; thus, any change in oral microbiota diversity is a risk factor (Le Bars et al., 2017).

This study showed that the rat oral microbiota consisted mainly of *Proteobacteria, Firmicutes*, and *Actinobacteria* at the phylum level, and that the administration of different types of antibiotics led to significant differences in their relative abundance (**Figures 4A**). *Proteobacteria, Firmicutes*, and *Actinobacteria* are the three most abundant bacterial phyla in the oral cavity of healthy humans (Verma et al., 2018), and changes in their abundance in rat oral cavity can reflect, to some extent, the internal conditions of the human body. A previous study found that most of the gut microbiota whose relative abundance increased with cefprozil treatment (*Lachnospiraceae, Blautia, Coprococcus, Roseburia*, and *Ruminococcus*) were from the *Firmicutes* phylum, which, along with *Bacteroides*, constitutes the majority of the human gut microbiota (Jarmusch et al., 2020). Our results showed that SP also increased the relative abundance of *Firmicutes* species. However, the specific mechanism is not precise.

The relative abundance of *Rodentibacter*, *Streptococcus*, *Veillonella*, and *Gemella* changed dramatically among all groups at the genus level (**Figure 2**). Previous studies have reported *Prevotella* and *Streptococcus* as the most abundant microbiota genera in human oral cavity (Rusthen et al., 2019), which is somewhat different from results of the present study. This distinction is related to the fact that humans and rats are different species. *Streptococcus* relative abundance changed little across all groups. However, whether antibiotics induce these alterations requires more *in vitro* testing. Another research found that as individuals aged, the relative abundance of *Lactobacillus* grew from 4% to 30%, which was connected with a progressive rise in the body’s digestive and absorptive capacity (Piekarska-Radzik and Klewicka, 2021). *Lactobacilli* are members of the *lactobacilli* family. They get their name from the fact that they ferment sugar and produce a lot of lactic acid. Its significance in maintaining human health and regulating immune function is well known. In our study, oral *Lactobacillus* relative abundance was increased in SP-treated rats. As a result, we postulate that various kinds of antibiotics impact the digestive capacity of rats, and that the same phenomena may potentially occur in people. *Rodentibacter*, which is a bacterial genus unique to the oral cavity of rats, was inhibited in all gavage groups (**Figure 2**) (Benga et al., 2019).

Our functional predictions suggested that the administration of different types of antibiotics led to changes in the metabolic pathways of oral microbiota in rats (**Figure 5**). Compared with the other drugs, MTZ caused more dramatic changes in the oral microbiota. **Figure 6** shows that α-linolenic acid metabolism, polyketide sugar unit biosynthesis, vitamin B6 metabolism, arginine proline metabolism, and starch and sucrose metabolism, all of which are normal microbial metabolic functions (helping digestion and nutrient absorption), increased significantly in the MTZ group (Deo and Deshmukh, 2019). In contrast, the functional pathways involved in vitamin B6 metabolism, ABC transporters(promote membrane formation and functional maintenance), and arginine and proline metabolism(protein synthesis) were reduced in the SP group; although these alterations in microbiota genetic metabolic pathways are not necessarily associated with oral disease, they deserve the attention of pathology researchers and prescribing clinicians (Xiao et al., 2020). Hierarchical cluster and principal coordinate analyses revealed some similarities or differences in the oral microbiota community composition of rats administered different types of antibiotics (**Figure 5**). As shown in **Figure 5**, the oral microbiota of rats can be divided into four significantly different taxa depending on the antibiotic treatment: oral microbiota in the SP and control groups were grouped closer together, suggesting that SP administration had a low effect on the normal oral microbiota ecology. This is confirmed by the finding that samples from the MTZ and AMX groups were grouped closer together, as shown by **Figure 5**. In contrast, oral microbiota samples from the control and SP groups were located in the other quadrants.

Oral immunoglobulin is an abundant Y-shaped protein secreted mainly by plasma cells and used by the immune system to identify and neutralize foreign substances, such as bacteria, viruses, and other pathogens. ELISA results showed the presence of IgG, IgM, and SIgA in the saliva samples of all rats. In the AMX group, IgG, IgM, and SIgA content significantly increased compared with that in the other groups (**Figures 2A–C**). As shown in **Figure 2**, the relative abundance of *Rothia* and *Haemophilus* in the AMX group was higher than that in the other groups; however, the relative abundance of *Acinetobacter, Staphylococcus*, and *Lactobacillus* in the SP group was higher than that in the other groups, and there was no corresponding change in rat salivary antibody concentrations.

In conclusion, our study shows that antibiotic-induced changes in the oral microbiota are not always linked to oral immunoglobulin content, and vice versa. The observed difference in the effects of the different classes of antibiotics on rat oral microbiota warrants further investigation, as it may be important during the selection of appropriate treatment for bacterial infections.

## Data Availability Statement

The datasets presented in this study can be found in online repositories. The names of the repository/repositories and accession number(s) can be found in the article/**Supplementary Material**.

## Ethics Statement

The Ethics Committee of the People’s Hospital of Leshan approved the design, agreements, and informed consent of our study (Leshan City, Sichuan Province, China). Ethics number: 2018-11-03-00089.

## Author Contributions

Conceived and designed the experiments: QC. Performed the experiments: XC, MS, PS, and FH. Contributed reagents/materials/analysis tools: XC, PS, FH, and QC. Wrote the paper: XC. All authors edited the manuscript and agreed with its final form. All authors read and approved the final manuscript.

## Funding

This study is supported by Zhejiang Medical and Health Science and Technology Project (No. 2020KY625), Fundamental Research Funds for the Central Universities (No.2020FZZX008-08).

## Conflict of Interest

The authors declare that the research was conducted in the absence of any commercial or financial relationships that could be construed as a potential conflict of interest.

## Publisher’s Note

All claims expressed in this article are solely those of the authors and do not necessarily represent those of their affiliated organizations, or those of the publisher, the editors and the reviewers. Any product that may be evaluated in this article, or claim that may be made by its manufacturer, is not guaranteed or endorsed by the publisher.

## Supplementary Material

Supplementary Figure 1 sobs index on OTU level

Supplementary Figure 2 shannon index on OTU level

Supplementary Figure 3 numbers of total OTU

Supplementary Figure 4 Community analysis pieplot on OTU level

Supplementary Figure 5 Stacked column plots representing comparison ofrelative abundance of bacterial taxa between all groups at class, order, family, andspecies.

Supplementary Figure 6 Correlations between bacteria and corresponding roups.

Supplementary Figure 7 Rarefaction analysis of bacterial 16S rRNA genesequences.

Supplementary Table 1 Antibiotic species and their target strains.

Supplementary Table 2 Samples read counts.

Supplementary Table 3 Comparison of significant bacterial genus in all groups

Supplementary Table 4 Multi-group difference statistics table (phylum level)

Supplementary Table 5 Multi-group difference statistics table (Genus level)
