# Supplementary figures and images for: Effects of Antibiotic Use on Saliva Antibody Content and Oral Microbiota in Sprague Dawley Rats
Source: Front Cell Infect Microbiol. 2022 Jan 31;12:721691. doi: 10.3389/fcimb.2022.721691 (PMC8843035; doi:10.3389/fcimb.2022.721691)

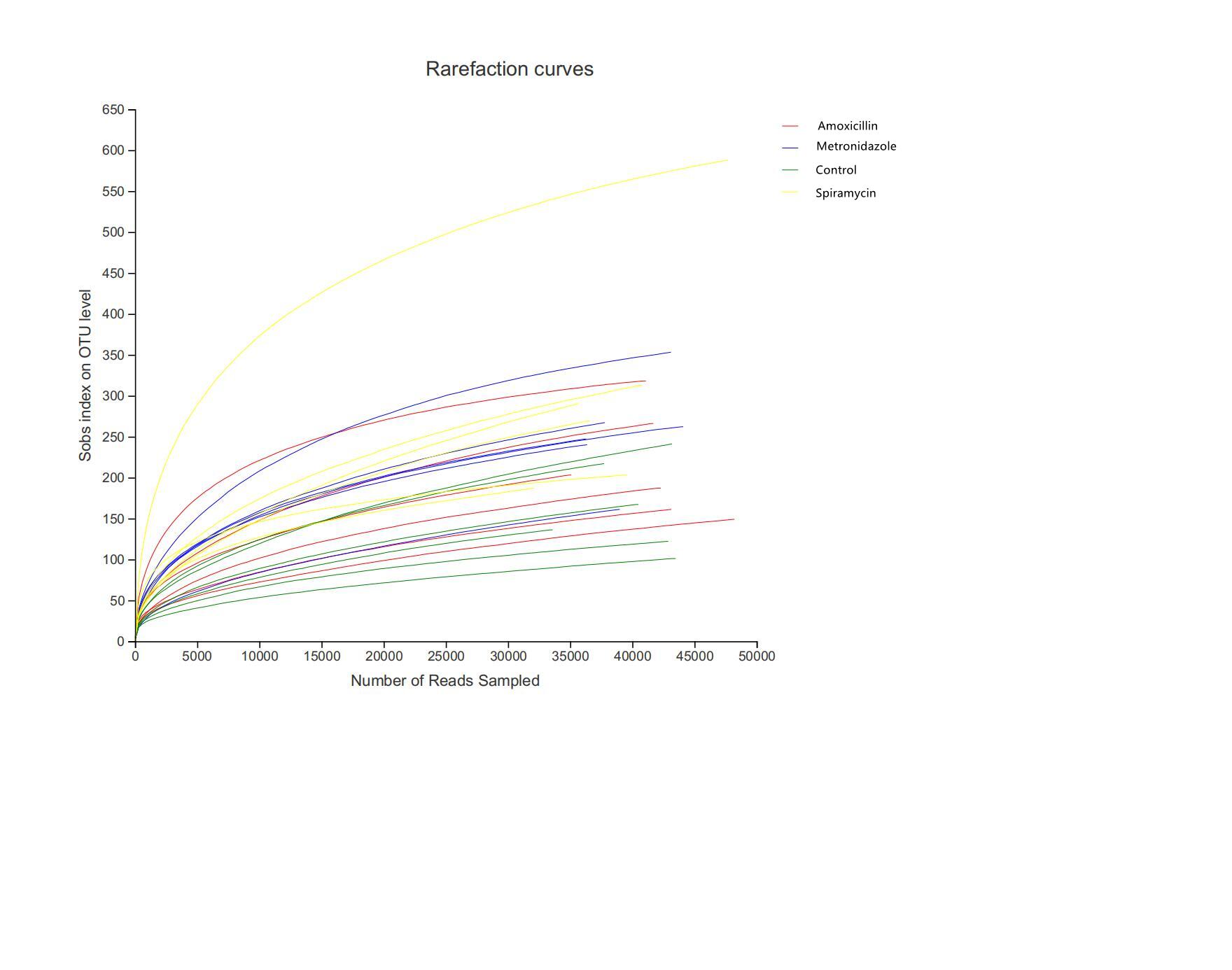

Supplement: Supplementary Figure 1 — sobs index on OTU level [file Image_1.jpeg]

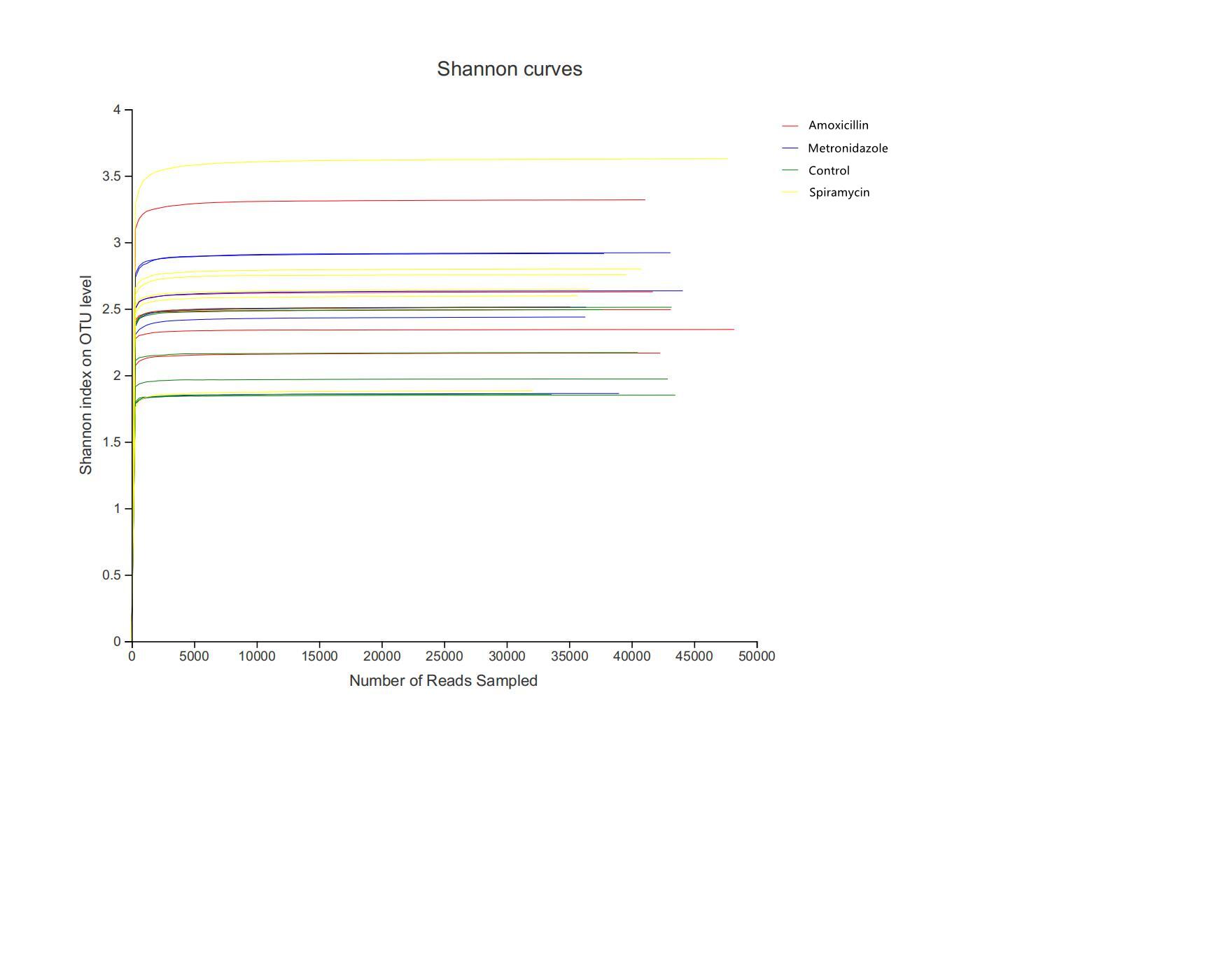

Supplement: Supplementary Figure 2 — shannon index on OTU level [file Image_2.jpeg]

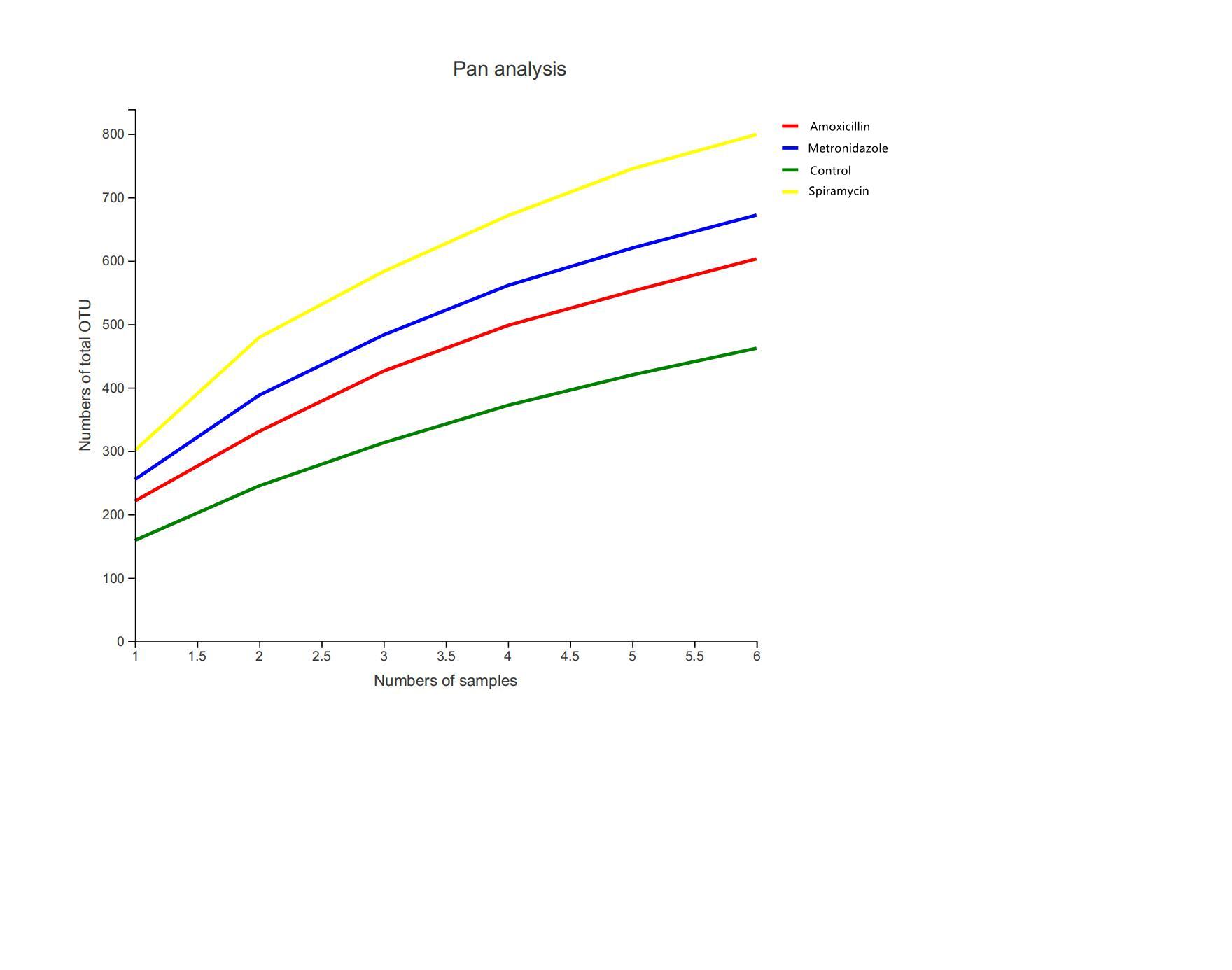

Supplement: Supplementary Figure 3 — numbers of total OTU [file Image_3.jpeg]

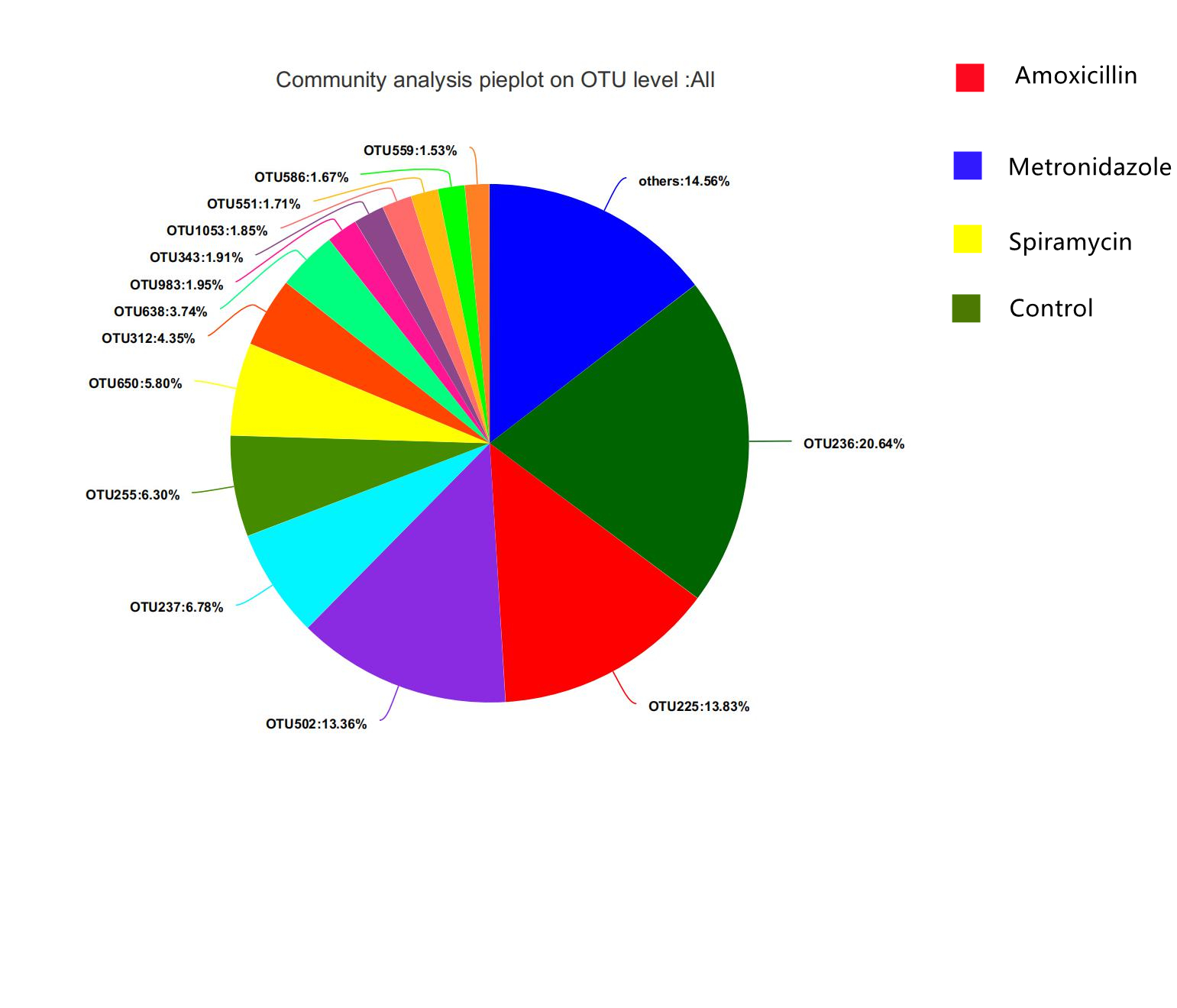

Supplement: Supplementary Figure 4 — Community analysis pieplot on OTU level [file Image_4.jpeg]

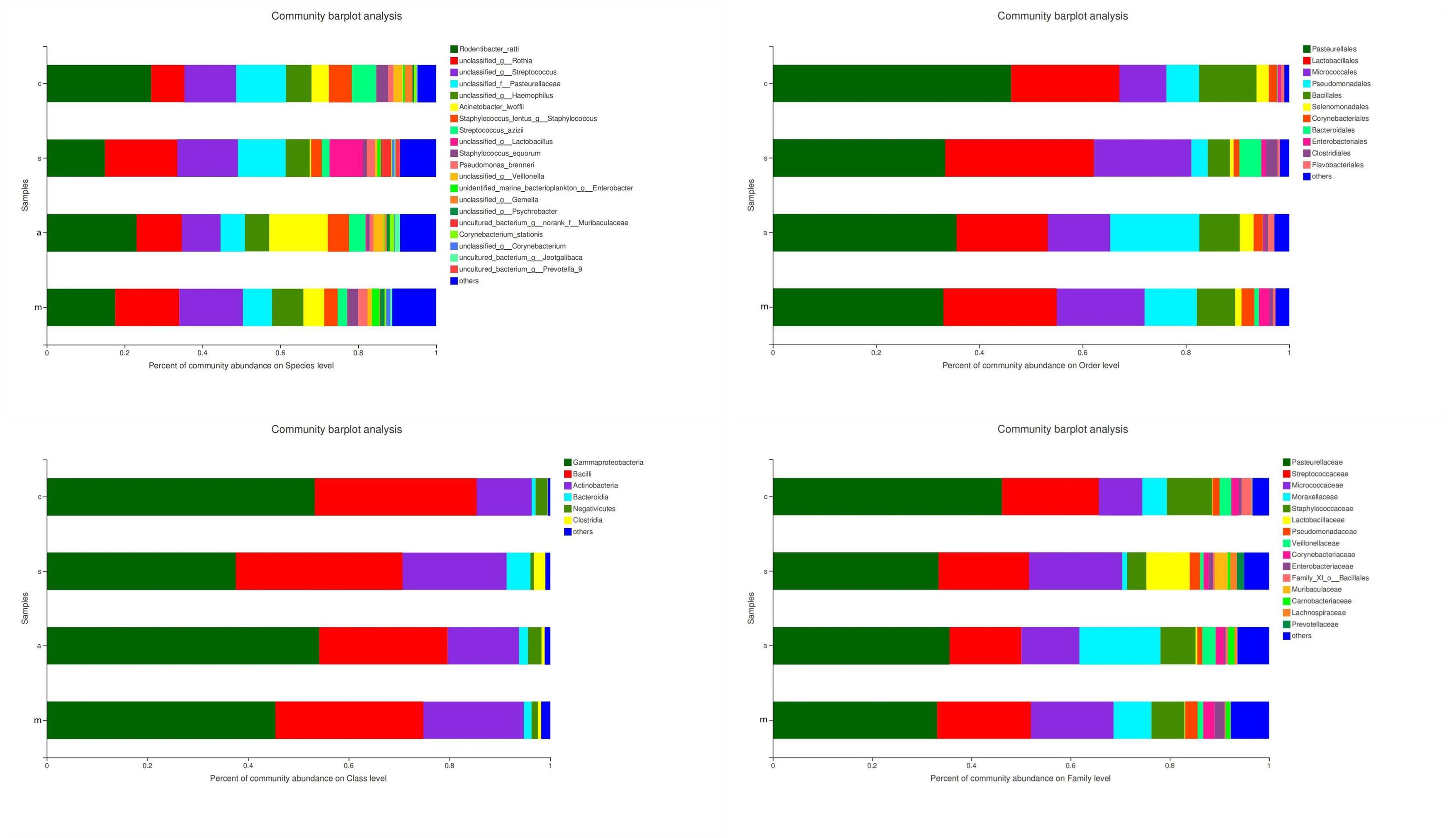

Supplement: Supplementary Figure 5 — Stacked column plots representing comparison ofrelative abundance of bacterial taxa between all groups at class, order, family, andspecies. [file Image_5.jpeg]

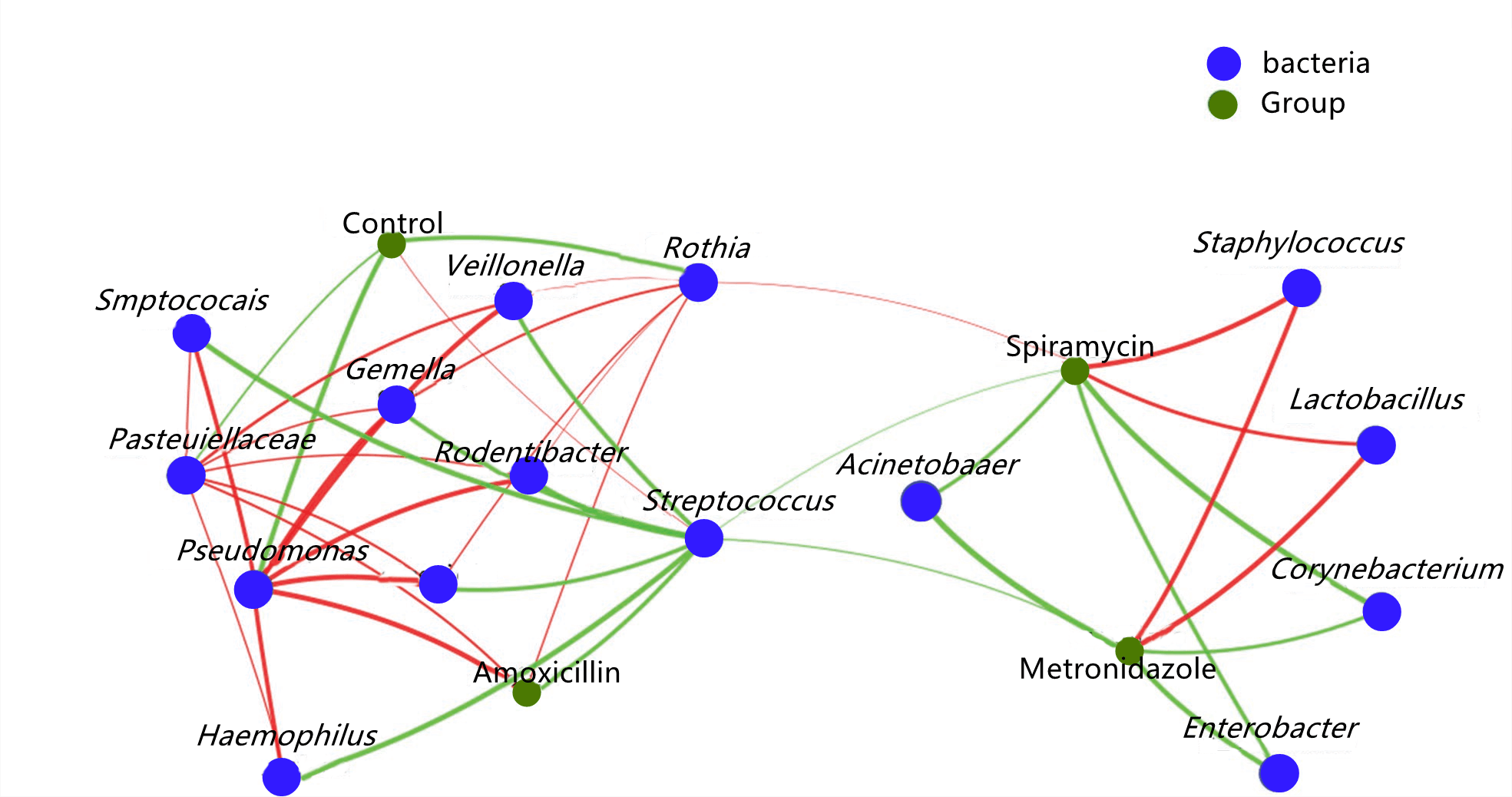

Supplement: Supplementary Figure 6 — Correlations between bacteria and corresponding roups. [file Image_6.png]

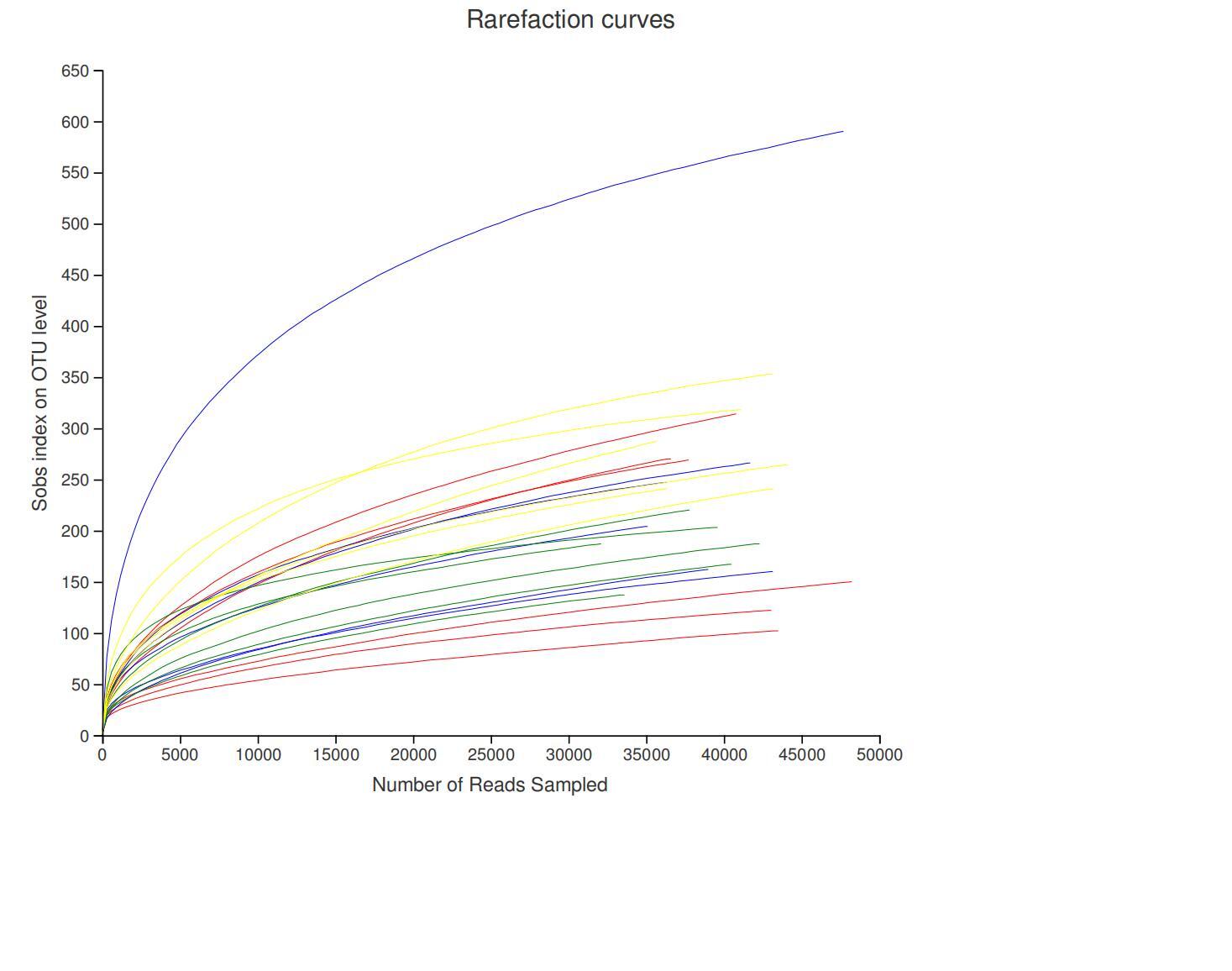

Supplement: Supplementary Figure 7 — Rarefaction analysis of bacterial 16S rRNA genesequences. [file Image_7.jpeg]
