# Supplementary material for: Effects of Antibiotic Use on Saliva Antibody Content and Oral Microbiota in Sprague Dawley Rats
Source: Front Cell Infect Microbiol. 2022 Jan 31;12:721691. doi: 10.3389/fcimb.2022.721691 (PMC8843035; doi:10.3389/fcimb.2022.721691)
Supplement: Supplementary Table 2 — Samples read counts. [file Table_2.docx]

| Antibiotic Abbr. Class Bacterial targets |
| --- |
| Amoxicillin AMX Penicillin Moderate spectrum,  Gram-positives    Spiramycin SP Spiramycin Gram-positives    Metronidazole MTZ Nitroimidazole Broad-spectrum,  anaerobes |
