# Supplementary material for: Effects of Antibiotic Use on Saliva Antibody Content and Oral Microbiota in Sprague Dawley Rats
Source: Front Cell Infect Microbiol. 2022 Jan 31;12:721691. doi: 10.3389/fcimb.2022.721691 (PMC8843035; doi:10.3389/fcimb.2022.721691)
Supplement: Supplementary Table 4 — Multi-group difference statistics table (phylum level) [file Table_4.docx]

| Sample group | Top four abundant genera | Composition ratio (%) |
| --- | --- | --- |
| Amoxicillin | Streptococcus | 20.25 |
|  | Rodentibarter | 18.34 |
|  | Rothia | 16.81 |
|  | Pasteuiellaceae | 10.82 |
| Metronidazole | Acinetobaaer | 19.56 |
|  | Staphylococcus | 13.20 |
|  | Streptococcus | 8.92 |
|  | Rodentibacter | 6.85 |
| Control | Rothia | 20.47 |
|  | Smptococais | 19.11 |
|  | Rodentibacter | 18.04 |
|  | Pasteuiellaceae | 10.69 |
| Spiramycin | Rodentlbacter | 36.05 |
|  | Streptococcus | 21.70 |
|  | Rothia | 10.73 |
|  | Pasteuiellaceae | 10.54 |
