# Supplementary material for: Effects of Antibiotic Use on Saliva Antibody Content and Oral Microbiota in Sprague Dawley Rats
Source: Front Cell Infect Microbiol. 2022 Jan 31;12:721691. doi: 10.3389/fcimb.2022.721691 (PMC8843035; doi:10.3389/fcimb.2022.721691)
Supplement: Supplementary Table 5 — Multi-group difference statistics table (Genus level) [file Table_5.docx]

Table.s4 Multi-group difference statistics table (phylum level)

| Species name | c-Mean(%) | c-Sd(%) | s-Mean(%) | s-Sd(%) | m-Mean(%) | sa-Sd(%) | a-Mean(%) | x-Sd(%) | P-value | Corrected pvalue |
| --- | --- | --- | --- | --- | --- | --- | --- | --- | --- | --- |
| Armatimonadetes | 0 | 0 | 0 | 0 | 0 | 0 | 0.005762 | 0.01279 | 0.09959 | 0.4335 |
| Spirochaetes | 0.0024 | 0.003086 | 0.01333 | 0.02091 | 0.006288 | 0.01194 | 0.6618 | 1.602 | 0.7273 | 0.7833 |
| Actinobacteria | 10.96 | 7.523 | 21.28 | 9.654 | 13.95 | 6.267 | 20.07 | 3.047 | 0.08656 | 0.4335 |
| Bacteroidetes | 0.7786 | 0.3016 | 4.117 | 8.251 | 1.76 | 1.756 | 1.536 | 1.889 | 0.943 | 0.943 |
| Patescibacteria | 0.0075 | 0.006463 | 0.09985 | 0.1526 | 0.01092 | 0.01311 | 0.03119 | 0.03091 | 0.3035 | 0.6092 |
| Tenericutes | 0.0119 | 0.01716 | 0.006379 | 0.006484 | 0.02411 | 0.04026 | 0.008145 | 0.01995 | 0.6576 | 0.7504 |
| Fusobacteria | 0.0018 | 0.004489 | 0.08272 | 0.1951 | 0.008937 | 0.02062 | 0.01974 | 0.03845 | 0.3115 | 0.6092 |
| Deinococcus | 0.0017 | 0.003315 | 0.002535 | 0.004064 | 0.0008832 | 0.002163 | 0.004104 | 0.004387 | 0.319 | 0.6092 |
| Verrucomicrobia | 0.0002 | 0.002163 | 0.005049 | 0.0098 | 0 | 0 | 0.003177 | 0.006505 | 0.2602 | 0.6092 |
| Proteobacteria | 53.19 | 5.201 | 38.8 | 13.39 | 55.59 | 11.75 | 46.05 | 4.503 | 0.03438 | 0.4335 |
| Chlamydiae | 0 | 0 | 0.0004767 | 0.001168 | 0.0009429 | 0.001472 | 0.0004588 | 0.001124 | 0.5163 | 0.7374 |
| Synergistetes | 0.0019 | 0.001995 | 0.01113 | 0.02343 | 0.008467 | 0.01382 | 0.2772 | 0.6698 | 0.6505 | 0.7504 |
| Chloroflexi | 0.0344 | 0.02674 | 0.05089 | 0.06887 | 0.04435 | 0.05133 | 0.1081 | 0.16 | 0.6077 | 0.7504 |
| Dependentiae | 0.0482 | 0.001122 | 0.0004605 | 0.001128 | 0 | 0 | 0 | 0 | 0.5531 | 0.7374 |
| Gemmatimon | 0.0033 | 0.004602 | 0.015 | 0.02051 | 0.001884 | 0.001475 | 0.003885 | 0.005659 | 0.2697 | 0.6092 |
| WPS-2 | 0.0024 | 0.003342 | 0.0011 | 0.001796 | 0.002932 | 0.00465 | 0.007942 | 0.01323 | 0.5521 | 0.7374 |
| Cloacimonetes | 0 | 0 | 0 | 0 | 0 | 0 | 0.004887 | 0.01197 | 0.3916 | 0.6092 |
| Acidobacteria | 0.0257 | 0.01558 | 0.03227 | 0.02841 | 0.01835 | 0.01362 | 0.02027 | 0.01782 | 0.3476 | 0.6092 |
| norank | 0 | 0 | 0.0008287 | 0.001285 | 0 | 0 | 0.004095 | 0.004723 | 0.0713 | 0.4335 |
| Halanaerobiaeota | 0 | 0 | 0 | 0 | 0 | 0 | 0.0007725 | 0.001892 | 0.3916 | 0.6092 |
| Elusimicrobia | 0 | 0 | 0 | 0 | 0 | 0 | 0.00733 | 0.01796 | 0.3916 | 0.6092 |
| Planctomycetes | 0.0016 | 0.001301 | 0 | 0 | 0 | 0 | 0.002088 | 0.003923 | 0.1084 | 0.4335 |
| Rokubacteria | 0 | 0 | 0.002134 | 0.004196 | 0 | 0 | 0 | 0 | 0.09959 | 0.4335 |
| Hydrogenedentes | 0 | 0 | 0 | 0 | 0 | 0 | 0.001222 | 0.002993 | 0.3916 | 0.6092 |
| Cyanobacteria | 0.0043 | 0.003688 | 0.03891 | 0.05242 | 0.001396 | 0.002447 | 0.00531 | 0.004201 | 0.002427 | 0.06795 |
| Nitrospirae | 0.0416 | 0.001082 | 0.002797 | 0.00685 | 0.0008101 | 0.001255 | 0.001323 | 0.002298 | 0.9231 | 0.943 |
