# Supplementary material for: Effects of Antibiotic Use on Saliva Antibody Content and Oral Microbiota in Sprague Dawley Rats
Source: Front Cell Infect Microbiol. 2022 Jan 31;12:721691. doi: 10.3389/fcimb.2022.721691 (PMC8843035; doi:10.3389/fcimb.2022.721691)
Supplement: Supplementary file 13 [file Table_6.docx]

Table.s5 Multi-group difference statistics table (Genus level)

| Species name | c-Mean(%) | c-Sd(%) | s-Mean(%) | s-Sd(%) | sa-Mean(%) | sa-Sd(%) | x-Mean(%) | x-Sd(%) | Pvalue | Corrected pvalue |
| --- | --- | --- | --- | --- | --- | --- | --- | --- | --- | --- |
| g__Acinetobacter | 4.758 | 6.487 | 0.421 | 0.5182 | 16.5 | 27.17 | 5.975 | 8.915 | 0.4528 | 0.7109 |
| g__Rothia | 8.44 | 6.13 | 19.29 | 9.82 | 11.43 | 7.751 | 16.65 | 4.313 | 0.1411 | 0.7109 |
| g__Macellibacteroides | 0.002103 | 0.001868 | 0.002498 | 0.005034 | 0.007201 | 0.01266 | 0.1954 | 0.4681 | 0.5297 | 0.7109 |
| g__Gemella | 1.933 | 3.149 | 0.3096 | 0.2866 | 0.5015 | 0.7126 | 0.21 | 0.1486 | 0.667 | 0.8075 |
| g__Blautia | 0.00827 | 0.01667 | 0.3166 | 0.5272 | 0.5361 | 1.312 | 0.03053 | 0.02642 | 0.1714 | 0.7109 |
| g__Lactobacillus | 0.1774 | 0.1948 | 7.665 | 14.05 | 0.3545 | 0.3755 | 0.1814 | 0.1285 | 0.6414 | 0.786 |
| g__Rodentibacter | 26.46 | 14.64 | 15.33 | 6.925 | 23.15 | 19.39 | 17.43 | 3.784 | 0.5385 | 0.7109 |
| g__Flavobacterium | 0.001717 | 0.002046 | 0.001211 | 0.001343 | 0.3553 | 0.7672 | 0.02139 | 0.03828 | 0.1802 | 0.7109 |
| g__Enterobacter | 0.4973 | 0.8356 | 0.661 | 0.9641 | 0.161 | 0.2416 | 1.777 | 2.468 | 0.3549 | 0.7109 |
| g__Streptococcus | 19.22 | 6.369 | 18.69 | 8 | 14.08 | 9.878 | 18.81 | 4.638 | 0.8463 | 0.8889 |
| g__norank_f__Muribaculaceae | 0.06866 | 0.1504 | 2.275 | 5.495 | 0.01424 | 0.02085 | 0.01752 | 0.0226 | 0.9044 | 0.921 |
| g__unclassified_o__Lactobacillales | 0.5671 | 0.3126 | 0.3723 | 0.261 | 0.6444 | 0.67 | 0.5077 | 0.5064 | 0.8447 | 0.8889 |
| g__Aerosphaera | 0.006409 | 0.007706 | 0.02779 | 0.04776 | 0.2439 | 0.4032 | 0.09412 | 0.1426 | 0.454 | 0.7109 |
| g__Haemophilus | 6.379 | 5.688 | 6.422 | 4.887 | 6.099 | 4.613 | 8.05 | 5.545 | 0.901 | 0.9194 |
| g__Aerococcus | 0.006764 | 0.006661 | 0.03221 | 0.04782 | 0.007754 | 0.008517 | 0.1342 | 0.1617 | 0.08641 | 0.7109 |
| g__Pseudomonas | 1.448 | 1.21 | 2.135 | 1.156 | 0.9211 | 1.386 | 2.436 | 0.746 | 0.1146 | 0.7109 |
| g__Brevibacterium | 0.1296 | 0.1558 | 0.06034 | 0.06763 | 0.09095 | 0.1485 | 0.1599 | 0.182 | 0.2973 | 0.7109 |
| g__Yaniella | 0.0885 | 0.1078 | 0.03152 | 0.03735 | 0.01974 | 0.02813 | 0.04101 | 0.02785 | 0.4969 | 0.7109 |
| g__Pseudochrobactrum | 0.02468 | 0.03771 | 0.004551 | 0.005513 | 0.05147 | 0.09054 | 0.03227 | 0.0352 | 0.3502 | 0.7109 |
| g__unclassified_f__Pasteurellaceae | 12.71 | 2.54 | 12.53 | 9.327 | 6.058 | 5.698 | 7.398 | 3.996 | 0.09647 | 0.7109 |
| g__Staphylococcus | 9.701 | 11.57 | 3.64 | 2.236 | 6.16 | 9.136 | 5.938 | 3.983 | 0.7739 | 0.8418 |
| g__Psychrobacter | 0.4013 | 0.1872 | 0.6396 | 0.806 | 1.046 | 1.489 | 2.041 | 1.881 | 0.1502 | 0.7109 |
| g__Sphaerochaeta | 0.002419 | 0.003086 | 0.002574 | 0.005036 | 0.005846 | 0.01191 | 0.6389 | 1.547 | 0.7255 | 0.8418 |
| g__Planomicrobium | 0.01434 | 0.008528 | 0.1354 | 0.06232 | 0.03705 | 0.01895 | 0.4234 | 0.4192 | 0.0003174 | 0.1587 |
| g__Allobaculum | 0.02384 | 0.05086 | 0.1594 | 0.2346 | 0.1809 | 0.3081 | 0.07575 | 0.1275 | 0.6245 | 0.7717 |
| g__Romboutsia | 0.0464 | 0.09308 | 0.1575 | 0.3729 | 0.01661 | 0.01615 | 0.02601 | 0.0326 | 0.7509 | 0.8418 |
| g__unclassified_f__Synergistaceae | 0.001229 | 0.001995 | 0.006806 | 0.01419 | 0.00593 | 0.009625 | 0.1596 | 0.3827 | 0.6757 | 0.8107 |
| g__Escherichia-Shigella | 0.04794 | 0.05619 | 0.1717 | 0.3753 | 0.201 | 0.2784 | 0.1699 | 0.2202 | 0.5024 | 0.7109 |
| g__Sphingobacterium | 0.08783 | 0.1269 | 0.0226 | 0.04223 | 0.343 | 0.6543 | 0.1002 | 0.07366 | 0.2064 | 0.7109 |
| g__Devosia | 0.04398 | 0.07886 | 0.005026 | 0.004153 | 0.05013 | 0.0865 | 0.03229 | 0.02619 | 0.3335 | 0.7109 |
| g__Veillonella | 2.252 | 2.386 | 0.6892 | 0.4869 | 2.608 | 4.179 | 1.137 | 0.7696 | 0.5614 | 0.7178 |
| g__unclassified_f__Lachnospiraceae | 0.005697 | 0.01285 | 0.5115 | 1.049 | 0.007065 | 0.01731 | 0.009303 | 0.008929 | 0.1824 | 0.7109 |
| g__Corynebacterium_1 | 1.277 | 1.509 | 0.4437 | 0.482 | 1.334 | 2.503 | 1.098 | 0.9593 | 0.6178 | 0.7711 |
| g__Stenotrophomonas | 0.1028 | 0.2151 | 0.00548 | 0.006348 | 0.7187 | 1.615 | 0.06891 | 0.1313 | 0.3171 | 0.7109 |
| g__Brachybacterium | 0.2178 | 0.2608 | 0.07263 | 0.06427 | 0.1247 | 0.2097 | 0.1424 | 0.1123 | 0.4729 | 0.7109 |
| g__Christensenellaceae_R-7_group | 0.001688 | 0.002629 | 0.03654 | 0.0859 | 0.007098 | 0.01271 | 0.1837 | 0.4379 | 0.4368 | 0.7109 |
| g__Solibacillus | 0.01104 | 0.02704 | 0.002447 | 0.005994 | 0.1308 | 0.2039 | 0.034 | 0.07242 | 0.1968 | 0.7109 |
| g__Prevotella_9 | 0.005387 | 0.007319 | 1.013 | 2.447 | 0.01764 | 0.04199 | 0.01996 | 0.03266 | 0.8355 | 0.885 |
| g__Desemzia | 0.0185 | 0.01299 | 0.1175 | 0.1182 | 0.0269 | 0.02477 | 0.3692 | 0.2617 | 0.002842 | 0.3098 |
| g__norank_f__Acidaminococcaceae | 0 | 0 | 0.005006 | 0.005785 | 0.00174 | 0.003041 | 0.1169 | 0.2767 | 0.06937 | 0.7109 |
| g__Jeotgalibaca | 0.01765 | 0.02749 | 0.3062 | 0.7385 | 1.212 | 2.247 | 0.5347 | 1.114 | 0.2978 | 0.7109 |
| g__Globicatella | 0.2824 | 0.2787 | 0.462 | 0.366 | 0.2449 | 0.3993 | 0.2677 | 0.1254 | 0.2651 | 0.7109 |
| g__Atopostipes | 0.03441 | 0.04158 | 0.06473 | 0.1076 | 0.04585 | 0.05782 | 0.06203 | 0.02914 | 0.3208 | 0.7109 |
| g__norank_f__M2PB4-65_termite_group | 0.002755 | 0.003406 | 0.003623 | 0.00756 | 0.00579 | 0.01418 | 0.1938 | 0.4725 | 0.7053 | 0.8259 |
| g__Moraxella | 0.03158 | 0.03431 | 0.02728 | 0.04784 | 0.01633 | 0.01612 | 0.07987 | 0.1387 | 0.8028 | 0.8618 |
| g__Facklamia | 0.08519 | 0.1358 | 0.08633 | 0.1141 | 0.1579 | 0.2806 | 0.1459 | 0.1175 | 0.299 | 0.7109 |
| g__norank_f__Synergistaceae | 0 | 0 | 0.004322 | 0.009257 | 0.002537 | 0.004194 | 0.1156 | 0.2821 | 0.4774 | 0.7109 |
| g__Desulfovibrio | 0.002594 | 0.002421 | 0.08572 | 0.1757 | 0.003194 | 0.004128 | 0.06806 | 0.1534 | 0.1814 | 0.7109 |
| g__Dietzia | 0.05112 | 0.07566 | 0.02406 | 0.01475 | 0.06386 | 0.08282 | 0.04969 | 0.04433 | 0.6761 | 0.8107 |
| g__Jeotgalicoccus | 0.1853 | 0.2347 | 0.1888 | 0.1955 | 0.8502 | 1.781 | 0.518 | 0.7221 | 0.6193 | 0.7711 |
| g__Enterococcus | 0.1865 | 0.2239 | 0.3571 | 0.3196 | 0.1977 | 0.1429 | 0.6454 | 0.779 | 0.1979 | 0.7109 |
| g__Actinomyces | 0.133 | 0.06607 | 0.1822 | 0.1704 | 0.2759 | 0.3381 | 0.2247 | 0.1957 | 0.8542 | 0.8913 |
| g__Brevundimonas | 0.07295 | 0.09564 | 0.02915 | 0.01087 | 0.2962 | 0.5086 | 0.1249 | 0.1084 | 0.4324 | 0.7109 |
| g__Bergeyella | 0.5304 | 0.3047 | 0.3981 | 0.3684 | 0.7372 | 1.289 | 0.3909 | 0.3231 | 0.8206 | 0.8732 |
| g__Enterorhabdus | 0.002092 | 0.003935 | 0.1178 | 0.2773 | 0.01162 | 0.0182 | 0.007486 | 0.009389 | 0.534 | 0.7109 |
| g__norank_f__Lachnospiraceae | 0.003414 | 0.005197 | 0.1255 | 0.2982 | 0.002073 | 0.003264 | 0.006329 | 0.008003 | 0.6827 | 0.8147 |
| g__Corynebacterium | 0.3201 | 0.4943 | 0.5719 | 0.6183 | 0.2605 | 0.1467 | 1.084 | 0.9617 | 0.1279 | 0.7109 |
| g__Bacteroides | 0.0037 | 0.003461 | 0.02371 | 0.03615 | 0.01422 | 0.01765 | 0.2472 | 0.5497 | 0.1542 | 0.7109 |
| g__Glutamicibacter | 0.04488 | 0.07127 | 0.02138 | 0.01934 | 0.04038 | 0.05447 | 0.1321 | 0.2319 | 0.2702 | 0.7109 |
| g__Chryseobacterium | 0.003833 | 0.00442 | 0.01229 | 0.008596 | 0.04893 | 0.06676 | 0.05094 | 0.05043 | 0.02402 | 0.632 |
| g__Rhodococcus | 0.07277 | 0.06476 | 0.0817 | 0.04997 | 0.05754 | 0.06885 | 0.124 | 0.04471 | 0.1679 | 0.7109 |
| g__Dubosiella | 0.008717 | 0.01375 | 0.01228 | 0.01655 | 0.1564 | 0.2666 | 0.04804 | 0.1023 | 0.531 | 0.7109 |
| g__Ruminococcaceae_UCG-014 | 0.003335 | 0.005961 | 0.1722 | 0.4039 | 0.00489 | 0.005141 | 0.005046 | 0.006968 | 0.6899 | 0.8157 |
| g__Bifidobacterium | 0.0129 | 0.02097 | 0.01408 | 0.01489 | 0.07105 | 0.07581 | 0.07579 | 0.07907 | 0.09406 | 0.7109 |
| g__Caproiciproducens | 0.002003 | 0.001954 | 0.01998 | 0.02699 | 0.005444 | 0.005611 | 0.02525 | 0.03537 | 0.1673 | 0.7109 |
| g__Ruminococcaceae_NK4A214_group | 0.0008832 | 0.002163 | 0.01409 | 0.03319 | 0.0004136 | 0.001013 | 0.03787 | 0.09277 | 0.8574 | 0.8913 |
| g__norank_f__Saccharimonadaceae | 0.006343 | 0.00581 | 0.03881 | 0.09202 | 0.006193 | 0.01093 | 0.01338 | 0.01077 | 0.2486 | 0.7109 |
| g__Myroides | 0.003268 | 0.008006 | 0.001748 | 0.004281 | 0.1026 | 0.2167 | 0.004076 | 0.006482 | 0.6382 | 0.784 |
| g__norank_f__JG30-KF-CM45 | 0.008747 | 0.008372 | 0.01318 | 0.01466 | 0.003394 | 0.003578 | 0.01635 | 0.01075 | 0.09499 | 0.7109 |
| g__Enteractinococcus | 0.02488 | 0.03519 | 0.02556 | 0.03851 | 0.01311 | 0.01348 | 0.03241 | 0.01579 | 0.2876 | 0.7109 |
| g__Lachnospiraceae_NK4A136_group | 0.0008171 | 0.002002 | 0.09963 | 0.244 | 0.0003966 | 0.0009714 | 0 | 0 | 0.7747 | 0.8418 |
| g__Petrimonas | 0.0007312 | 0.001135 | 0 | 0 | 0.001269 | 0.002097 | 0.03665 | 0.08978 | 0.495 | 0.7109 |
| g__norank_f__Bacteroidetes_vadinHA17 | 0.0003857 | 0.0009446 | 0.002447 | 0.005994 | 0.0117 | 0.02053 | 0.06926 | 0.1672 | 0.5237 | 0.7109 |
| g__Ruminiclostridium_6 | 0.0007541 | 0.001174 | 0.08809 | 0.2158 | 0 | 0 | 0 | 0 | 0.2871 | 0.7109 |
| g__Clostridium_sensu_stricto_1 | 0.03207 | 0.06875 | 0.006036 | 0.00812 | 0.004938 | 0.006943 | 0.05052 | 0.1129 | 0.8526 | 0.8913 |
| g__Prevotellaceae_UCG-003 | 0.0004086 | 0.001001 | 0.06327 | 0.155 | 0 | 0 | 0 | 0 | 0.5531 | 0.7109 |
| g__Ruminococcaceae_UCG-013 | 0.0004086 | 0.001001 | 0.08398 | 0.2044 | 0.0008832 | 0.002163 | 0.004167 | 0.01021 | 0.8225 | 0.8732 |
| g__Comamonas | 0.008565 | 0.009978 | 0.001398 | 0.003425 | 0.06921 | 0.1266 | 0.02263 | 0.04435 | 0.09937 | 0.7109 |
| g__unclassified_f__Aerococcaceae | 0.00858 | 0.02102 | 0.007214 | 0.01767 | 0.02372 | 0.02238 | 0.02682 | 0.04238 | 0.3811 | 0.7109 |
| g__unclassified_o__Micrococcales | 0.006406 | 0.0052 | 0.004867 | 0.006423 | 0.01137 | 0.01621 | 0.01224 | 0.01221 | 0.793 | 0.8564 |
| g__[Eubacterium]_coprostanoligenes_group | 0 | 0 | 0.03041 | 0.0745 | 0.0009133 | 0.001424 | 0.00154 | 0.00281 | 0.505 | 0.7109 |
| g__Paracoccus | 0.01016 | 0.01398 | 0.002107 | 0.002835 | 0.007033 | 0.01204 | 0.009445 | 0.00665 | 0.3343 | 0.7109 |
| g__unclassified_o__Clostridiales | 0.001688 | 0.002629 | 0 | 0 | 0 | 0 | 0.03257 | 0.05378 | 0.08595 | 0.7109 |
| g__Fusobacterium | 0 | 0 | 0.01002 | 0.01955 | 0.008495 | 0.02081 | 0.01161 | 0.02844 | 0.1142 | 0.7109 |
| g__Prevotellaceae_UCG-001 | 0.0017 | 0.002637 | 0.0409 | 0.1002 | 0 | 0 | 0.001692 | 0.002635 | 0.505 | 0.7109 |
| g__norank_f__norank_o__Actinomarinales | 0.007494 | 0.009788 | 0.023 | 0.04844 | 0.0007932 | 0.001943 | 0.009146 | 0.01067 | 0.2132 | 0.7109 |
| g__Cetobacterium | 0.0004582 | 0.001122 | 0.02806 | 0.06748 | 0.0004416 | 0.001082 | 0.006951 | 0.009487 | 0.2988 | 0.7109 |
| g__norank_f__Bradymonadaceae | 0.02641 | 0.06236 | 0.000428 | 0.001048 | 0.0005198 | 0.001273 | 0.001225 | 0.001976 | 0.6042 | 0.761 |
| g__Ralstonia | 0.01208 | 0.0137 | 0.01854 | 0.02011 | 0.01273 | 0.02178 | 0.04122 | 0.02231 | 0.08651 | 0.7109 |
| g__norank_f__Bacillaceae | 0.01291 | 0.02017 | 0.01392 | 0.0189 | 0.01394 | 0.0134 | 0.03605 | 0.03701 | 0.178 | 0.7109 |
| g__Pedobacter | 0.008119 | 0.01207 | 0.01124 | 0.007769 | 0.01057 | 0.008854 | 0.01505 | 0.008889 | 0.2319 | 0.7109 |
| g__Mycoplasma | 0.01191 | 0.01716 | 0.003932 | 0.005404 | 0.02325 | 0.04031 | 0 | 0 | 0.2225 | 0.7109 |
| g__Neisseria | 0.0004582 | 0.001122 | 0.02573 | 0.06185 | 0.0004416 | 0.001082 | 0.005418 | 0.01327 | 0.8668 | 0.8936 |
| g__norank_f__Propionibacteriaceae | 0.001269 | 0.002181 | 0.001501 | 0.002333 | 0.002652 | 0.003305 | 0.02618 | 0.06143 | 0.87 | 0.895 |
| g__Prevotellaceae_Ga6A1_group | 0.001634 | 0.004003 | 0.02671 | 0.05599 | 0 | 0 | 0.002273 | 0.005567 | 0.2093 | 0.7109 |
| g__unclassified_f__Sphingobacteriaceae | 0.001667 | 0.002981 | 0.0009211 | 0.002256 | 0.03923 | 0.08431 | 0.003383 | 0.002811 | 0.2514 | 0.7109 |
| g__Paenochrobactrum | 0.004546 | 0.003037 | 0.00116 | 0.001853 | 0.02468 | 0.05169 | 0.006725 | 0.004186 | 0.1505 | 0.7109 |
| g__norank_f__Desulfovibrionaceae | 0.0008832 | 0.002163 | 0.05768 | 0.1413 | 0 | 0 | 0 | 0 | 0.5531 | 0.7109 |
| g__Turicibacter | 0.02697 | 0.05497 | 0.004574 | 0.005478 | 0.004785 | 0.005059 | 0.01161 | 0.0163 | 0.7054 | 0.8259 |
| g__Marvinbryantia | 0.004903 | 0.01201 | 0.06446 | 0.1338 | 0.0008832 | 0.002163 | 0.005014 | 0.01096 | 0.4201 | 0.7109 |
| g__Ruminiclostridium | 0.0006911 | 0.001693 | 0.01363 | 0.02363 | 0.002395 | 0.004797 | 0.0101 | 0.007843 | 0.01381 | 0.555 |
| g__Paenalcaligenes | 0.003483 | 0.003934 | 0.003689 | 0.006712 | 0.01096 | 0.02134 | 0.008343 | 0.007173 | 0.537 | 0.7109 |
| g__Ercella | 0.0004582 | 0.001122 | 0.001875 | 0.00339 | 0.002593 | 0.004395 | 0.04398 | 0.1077 | 0.8223 | 0.8732 |
| g__Leptotrichia | 0.001375 | 0.003367 | 0.04464 | 0.1081 | 0 | 0 | 0.001181 | 0.001958 | 0.4756 | 0.7109 |
| g__norank_f__Xanthobacteraceae | 0.01132 | 0.01139 | 0.01231 | 0.004027 | 0.01159 | 0.006025 | 0.01186 | 0.004046 | 0.4425 | 0.7109 |
| g__Gemmobacter | 0.009075 | 0.01614 | 0 | 0 | 0.02893 | 0.06214 | 0.001314 | 0.001449 | 0.3796 | 0.7109 |
| g__Lactococcus | 0.00374 | 0.00424 | 0.01861 | 0.02372 | 0.001941 | 0.003615 | 0.009083 | 0.008888 | 0.2366 | 0.7109 |
| g__Salinicoccus | 0.001196 | 0.001323 | 0.0105 | 0.008661 | 0.004657 | 0.005073 | 0.01694 | 0.01175 | 0.009454 | 0.5252 |
| g__norank_f__Sphingobacteriaceae | 0.01837 | 0.02806 | 0.003261 | 0.006878 | 0.006769 | 0.01658 | 0.002475 | 0.003113 | 0.6251 | 0.7717 |
| g__Faecalibaculum | 0.00289 | 0.001961 | 0.01155 | 0.01858 | 0.00958 | 0.02099 | 0.002872 | 0.002853 | 0.7533 | 0.8418 |
| g__Leucobacter | 0.01608 | 0.02402 | 0.00497 | 0.005572 | 0.05542 | 0.09576 | 0.01563 | 0.01067 | 0.4605 | 0.7109 |
| g__Prevotella | 0.004881 | 0.008468 | 0.04836 | 0.0477 | 0.01125 | 0.02242 | 0.02319 | 0.02309 | 0.213 | 0.7109 |
| g__norank_f__norank_o__Coriobacteriales | 0 | 0 | 0.04125 | 0.101 | 0 | 0 | 0.001136 | 0.002784 | 0.5531 | 0.7109 |
| g__Longispora | 0.008902 | 0.01481 | 0.02503 | 0.05083 | 0.001603 | 0.002888 | 0.009029 | 0.01796 | 0.5594 | 0.7172 |
| g__unclassified_f__Ruminococcaceae | 0.002043 | 0.005004 | 0.02029 | 0.04272 | 0.001269 | 0.002097 | 0.02016 | 0.04067 | 0.5383 | 0.7109 |
| g__Capnocytophaga | 0.0004416 | 0.001082 | 0.03316 | 0.08122 | 0.001338 | 0.001479 | 0.003096 | 0.007584 | 0.65 | 0.7946 |
| g__Paludibacter | 0 | 0 | 0.001449 | 0.00256 | 0 | 0 | 0.03133 | 0.07563 | 0.2076 | 0.7109 |
| g__norank_f__norank_o__Chloroplast | 0.004613 | 0.003688 | 0.02313 | 0.01645 | 0.001396 | 0.002447 | 0.003334 | 0.003523 | 0.002443 | 0.3098 |
| g__Ileibacterium | 0.0008667 | 0.001346 | 0.001907 | 0.004671 | 0.01808 | 0.02997 | 0.005506 | 0.01349 | 0.7972 | 0.8591 |
| g__Aquamicrobium | 0.01567 | 0.02904 | 0.006847 | 0.00543 | 0.01374 | 0.0223 | 0.01364 | 0.01345 | 0.6917 | 0.8157 |
| g__Sporosarcina | 0.005244 | 0.005672 | 0.005915 | 0.006737 | 0.0187 | 0.03261 | 0.01091 | 0.007361 | 0.4493 | 0.7109 |
| g__Mycobacterium | 0.003971 | 0.005174 | 0.01574 | 0.03165 | 0.002708 | 0.005301 | 0.004127 | 0.003204 | 0.6194 | 0.7711 |
| g__Candidatus_Saccharimonas | 0.001239 | 0.001361 | 0.05658 | 0.1326 | 0.002107 | 0.002967 | 0.002806 | 0.002699 | 0.4949 | 0.7109 |
| g__Ornatilinea | 0.0007713 | 0.001889 | 0.0011 | 0.001796 | 0.003393 | 0.005442 | 0.05371 | 0.1246 | 0.3684 | 0.7109 |
| g__Planococcus | 0.002999 | 0.003917 | 0.002492 | 0.003023 | 0.00372 | 0.003653 | 0.03901 | 0.02731 | 0.003727 | 0.3098 |
| g__norank_f__norank_o__norank_c__Subgroup_6 | 0.007385 | 0.005084 | 0.01988 | 0.02306 | 0.008944 | 0.006799 | 0.008707 | 0.009118 | 0.4897 | 0.7109 |
| g__Luteimonas | 0.006785 | 0.009549 | 0.003053 | 0.005064 | 0.005738 | 0.008896 | 0.02903 | 0.02777 | 0.1232 | 0.7109 |
| g__norank_f__Prolixibacteraceae | 0.001229 | 0.001995 | 0.0003496 | 0.0008563 | 0.003505 | 0.007436 | 0.02075 | 0.0497 | 0.8032 | 0.8618 |
| g__Ruminococcus_1 | 0.0004086 | 0.001001 | 0.0219 | 0.05097 | 0 | 0 | 0.0003788 | 0.0009279 | 0.439 | 0.7109 |
| g__Bradyrhizobium | 0.004135 | 0.002933 | 0.00927 | 0.003465 | 0.003529 | 0.002282 | 0.008975 | 0.003352 | 0.01088 | 0.5438 |
| g__Parasutterella | 0.0008171 | 0.002002 | 0.01659 | 0.03817 | 0 | 0 | 0.0007576 | 0.001856 | 0.439 | 0.7109 |
| g__Lentimicrobium | 0 | 0 | 0.001875 | 0.00339 | 0.005299 | 0.01298 | 0.008184 | 0.01758 | 0.2888 | 0.7109 |
| g__norank_f__Ruminococcaceae | 0.0008832 | 0.002163 | 0.01328 | 0.03254 | 0.0004136 | 0.001013 | 0.009745 | 0.02278 | 0.8976 | 0.9178 |
| g__unclassified_f__Rhizobiaceae | 0.003467 | 0.006398 | 0.001271 | 0.002247 | 0.01309 | 0.01944 | 0.007706 | 0.009704 | 0.2442 | 0.7109 |
| g__DNF00809 | 0.0004086 | 0.001001 | 0.02412 | 0.05908 | 0.0004231 | 0.001036 | 0.0007576 | 0.001856 | 0.9979 | 0.9979 |
| g__[Eubacterium]_xylanophilum_group | 0 | 0 | 0.02211 | 0.05186 | 0 | 0 | 0.00231 | 0.003522 | 0.05839 | 0.7109 |
| g__Sphingomonas | 0.004957 | 0.008606 | 0.01162 | 0.01337 | 0.003909 | 0.00268 | 0.002007 | 0.001779 | 0.1295 | 0.7109 |
| g__Streptomyces | 0.004274 | 0.005111 | 0.01288 | 0.01542 | 0.0004997 | 0.001224 | 0.00844 | 0.005762 | 0.01836 | 0.5729 |
| g__Ruminiclostridium_9 | 0.003205 | 0.006886 | 0.01588 | 0.03549 | 0.0004231 | 0.001036 | 0.004253 | 0.006453 | 0.6904 | 0.8157 |
| g__Carnobacterium | 0.002912 | 0.003829 | 0.008793 | 0.01929 | 0.0004231 | 0.001036 | 0.01107 | 0.01904 | 0.5003 | 0.7109 |
| g__unclassified_f__Rhodobacteraceae | 0.0009164 | 0.002245 | 0.01518 | 0.03199 | 0.002163 | 0.004241 | 0.007433 | 0.01469 | 0.5742 | 0.7305 |
| g__Erysipelotrichaceae_UCG-003 | 0.003677 | 0.009007 | 0.008194 | 0.01679 | 0 | 0 | 0.002652 | 0.006495 | 0.5163 | 0.7109 |
| g__Nocardiopsis | 0.001325 | 0.003245 | 0.001679 | 0.002943 | 0.002979 | 0.003618 | 0.01872 | 0.03574 | 0.4341 | 0.7109 |
| g__Adlercreutzia | 0.0008998 | 0.001394 | 0.01189 | 0.02911 | 0.001403 | 0.00228 | 0.001755 | 0.003313 | 0.962 | 0.9737 |
| g__Actinomadura | 0.00175 | 0.002158 | 0.007966 | 0.01568 | 0.0003966 | 0.0009714 | 0.003804 | 0.004672 | 0.2921 | 0.7109 |
| g__Microbacterium | 0.005671 | 0.01028 | 0.005537 | 0.003601 | 0.005484 | 0.008857 | 0.006657 | 0.006554 | 0.4766 | 0.7109 |
| g__Raineyella | 0.001661 | 0.00202 | 0.00567 | 0.005852 | 0.0121 | 0.02548 | 0.002466 | 0.002727 | 0.6235 | 0.7717 |
| g__norank_f__norank_o__Candidatus_Pacebacteria | 0 | 0 | 0.0003496 | 0.0008563 | 0.0004136 | 0.001013 | 0.01344 | 0.03292 | 0.7747 | 0.8418 |
| g__Dysgonomonas | 0 | 0 | 0.0003496 | 0.0008563 | 0.0008271 | 0.002026 | 0.013 | 0.03075 | 0.4764 | 0.7109 |
| g__Nocardia | 0 | 0 | 0.009671 | 0.02369 | 0.0008551 | 0.001326 | 0.003096 | 0.007584 | 0.5832 | 0.7364 |
| g__norank_f__Erysipelotrichaceae | 0.0004086 | 0.001001 | 0.007452 | 0.01155 | 0.009245 | 0.02145 | 0 | 0 | 0.3956 | 0.7109 |
| g__Prevotella_7 | 0 | 0 | 0.01554 | 0.03576 | 0 | 0 | 0 | 0 | 0.09959 | 0.7109 |
| g__Saccharopolyspora | 0.002543 | 0.002288 | 0.005671 | 0.008771 | 0 | 0 | 0.004842 | 0.00746 | 0.07807 | 0.7109 |
| g__norank_f__norank_o__norank_c__AD3 | 0.008925 | 0.01734 | 0.002329 | 0.002955 | 0.005348 | 0.01002 | 0.004259 | 0.005781 | 0.9657 | 0.9755 |
| g__BRH-c57 | 0.001262 | 0.002235 | 0.003072 | 0.003862 | 0.008832 | 0.02163 | 0.001403 | 0.003437 | 0.3623 | 0.7109 |
| g__Acidothermus | 0.004374 | 0.001565 | 0.001202 | 0.002945 | 0.003177 | 0.003112 | 0.01321 | 0.01301 | 0.1169 | 0.7109 |
| g__Hydrogenispora | 0.001713 | 0.003194 | 0.001604 | 0.001841 | 0 | 0 | 0.01477 | 0.03619 | 0.3019 | 0.7109 |
| g__Ruminococcaceae_UCG-008 | 0.0008171 | 0.002002 | 0.01404 | 0.02613 | 0.0004416 | 0.001082 | 0.0003862 | 0.0009461 | 0.7334 | 0.8418 |
| g__Selenomonas_3 | 0 | 0 | 0.01842 | 0.04512 | 0.0004416 | 0.001082 | 0.002322 | 0.005688 | 0.7747 | 0.8418 |
| g__norank_f__norank_o__Gaiellales | 0.007067 | 0.007851 | 0.003421 | 0.004676 | 0.001416 | 0.001571 | 0.007202 | 0.00495 | 0.2245 | 0.7109 |
| g__Alloprevotella | 0.001667 | 0.002981 | 0.01129 | 0.02537 | 0.0008197 | 0.001271 | 0.002414 | 0.003602 | 0.8616 | 0.8927 |
| g__norank_f__norank_o__Gastranaerophilales | 0 | 0 | 0.01578 | 0.03749 | 0 | 0 | 0 | 0 | 0.09959 | 0.7109 |
| g__Paenibacillus | 0.0008667 | 0.001346 | 0.0004767 | 0.001168 | 0.00104 | 0.002547 | 0.01113 | 0.01831 | 0.4514 | 0.7109 |
| g__Ruminococcaceae_UCG-010 | 0 | 0 | 0.000428 | 0.001048 | 0.0008271 | 0.002026 | 0.01222 | 0.02993 | 0.7747 | 0.8418 |
| g__norank_f__67-14 | 0.001617 | 0.002103 | 0.01437 | 0.02075 | 0.003142 | 0.00271 | 0.002546 | 0.002291 | 0.2916 | 0.7109 |
| g__Treponema_2 | 0 | 0 | 0.01005 | 0.01523 | 0 | 0 | 0.006984 | 0.01577 | 0.09881 | 0.7109 |
| g__Oceanisphaera | 0.003042 | 0.003837 | 0 | 0 | 0.001692 | 0.004145 | 0.009349 | 0.01953 | 0.3019 | 0.7109 |
| g__Alkanindiges | 0.003974 | 0.009735 | 0.001338 | 0.001474 | 0.003675 | 0.00579 | 0.004402 | 0.007064 | 0.6599 | 0.8016 |
| g__Eubacterium | 0.001275 | 0.00209 | 0.0006991 | 0.001713 | 0 | 0 | 0.01053 | 0.01886 | 0.02062 | 0.5729 |
| g__Sporobacter | 0.0004086 | 0.001001 | 0.0007504 | 0.001166 | 0 | 0 | 0.01792 | 0.0392 | 0.1683 | 0.7109 |
| g__norank_f__AKYG1722 | 0.003417 | 0.004056 | 0.005879 | 0.00935 | 0.0008366 | 0.001296 | 0.002562 | 0.003996 | 0.7778 | 0.8418 |
| g__Lachnospiraceae_UCG-001 | 0.0004582 | 0.001122 | 0.01126 | 0.02636 | 0 | 0 | 0.0007658 | 0.001187 | 0.4661 | 0.7109 |
| g__Alistipes | 0.004593 | 0.008836 | 0.01023 | 0.02276 | 0 | 0 | 0.002298 | 0.00356 | 0.3453 | 0.7109 |
| g__Cellvibrio | 0.003664 | 0.003374 | 0.002473 | 0.003171 | 0.01266 | 0.02538 | 0.007055 | 0.007272 | 0.5399 | 0.7109 |
| g__unclassified_f__Eggerthellaceae | 0.0004086 | 0.001001 | 0.02143 | 0.05016 | 0.002268 | 0.003114 | 0.0004588 | 0.001124 | 0.4443 | 0.7109 |
| g__[Ruminococcus]_torques_group | 0.002451 | 0.006005 | 0.01524 | 0.0337 | 0.001766 | 0.004327 | 0.002273 | 0.005567 | 0.5514 | 0.7109 |
| g__Anaerofilum | 0 | 0 | 0 | 0 | 0.001654 | 0.004052 | 0.01425 | 0.03491 | 0.5531 | 0.7109 |
| g__Pelagibacterium | 0.004267 | 0.005672 | 0.001562 | 0.001817 | 0.01315 | 0.02386 | 0.005036 | 0.003743 | 0.4076 | 0.7109 |
| g__Porphyromonas | 0.0008998 | 0.001394 | 0.01289 | 0.03159 | 0.0004416 | 0.001082 | 0.001548 | 0.003792 | 0.8915 | 0.9135 |
| g__norank_f__Demequinaceae | 0.001687 | 0.002138 | 0.004291 | 0.01051 | 0.007686 | 0.01619 | 0.006542 | 0.008406 | 0.4705 | 0.7109 |
| g__Conexibacter | 0.006244 | 0.004245 | 0 | 0 | 0 | 0 | 0.008465 | 0.007057 | 0.004956 | 0.3098 |
| g__Bacillus | 0.007463 | 0.006729 | 0.009386 | 0.01248 | 0.002256 | 0.002737 | 0.006311 | 0.008722 | 0.6863 | 0.8157 |
| g__Prevotellaceae_NK3B31_group | 0.006128 | 0.01501 | 0.01519 | 0.03379 | 0.0008832 | 0.002163 | 0.0003788 | 0.0009279 | 0.7778 | 0.8418 |
| g__norank_f__Clostridiales_vadinBB60_group | 0 | 0 | 0.0003496 | 0.0008563 | 0 | 0 | 0.01344 | 0.03292 | 0.5531 | 0.7109 |
| g__Thermobifida | 0.003945 | 0.006262 | 0.00688 | 0.01416 | 0.0003966 | 0.0009714 | 0.002481 | 0.004013 | 0.3768 | 0.7109 |
| g__Aeromonas | 0.006229 | 0.008817 | 0.008153 | 0.01082 | 0.0004136 | 0.001013 | 0.007123 | 0.007343 | 0.121 | 0.7109 |
| g__Burkholderia-Caballeronia-Paraburkholderia | 0.0004416 | 0.001082 | 0.007914 | 0.01377 | 0.002236 | 0.001888 | 0.00774 | 0.006487 | 0.1301 | 0.7109 |
| g__Cutibacterium | 0.003124 | 0.005425 | 0.002674 | 0.003106 | 0.001586 | 0.003886 | 0.008188 | 0.01761 | 0.7725 | 0.8418 |
| g__norank_f__Rikenellaceae | 0.0003857 | 0.0009446 | 0.0004767 | 0.001168 | 0 | 0 | 0.01222 | 0.02993 | 0.7747 | 0.8418 |
| g__norank_f__norank_o__SBR1031 | 0.001196 | 0.001323 | 0.005593 | 0.0137 | 0.002649 | 0.00649 | 0.002888 | 0.003831 | 0.6606 | 0.8016 |
| g__Thermocrispum | 0.00347 | 0.00525 | 0.005831 | 0.01161 | 0 | 0 | 0.001566 | 0.002814 | 0.2388 | 0.7109 |
| g__Nocardioides | 0.002129 | 0.003094 | 0.005226 | 0.0057 | 0.0009164 | 0.001439 | 0.003248 | 0.002946 | 0.3694 | 0.7109 |
| g__Delftia | 0.002188 | 0.002647 | 0.001817 | 0.003441 | 0 | 0 | 0.006111 | 0.005993 | 0.03967 | 0.7109 |
| g__unclassified_f__Propionibacteriaceae | 0.006945 | 0.01701 | 0.001049 | 0.002569 | 0.001755 | 0.002046 | 0.00158 | 0.001972 | 0.6692 | 0.8082 |
| g__norank_f__norank_o__IMCC26256 | 0.003059 | 0.004101 | 0.003157 | 0.002981 | 0.003081 | 0.001791 | 0.002014 | 0.002731 | 0.699 | 0.8224 |
| g__Ornithinicoccus | 0.002986 | 0.003054 | 0.00375 | 0.006781 | 0.0005198 | 0.001273 | 0.004567 | 0.005515 | 0.3495 | 0.7109 |
| g__norank_f__norank_o__norank_c__Actinobacteria | 0.002534 | 0.00243 | 0.0006991 | 0.001713 | 0.001639 | 0.002542 | 0.004255 | 0.003826 | 0.1642 | 0.7109 |
| g__norank_f__Spirochaetaceae | 0 | 0 | 0.0006991 | 0.001713 | 0 | 0 | 0.009774 | 0.02394 | 0.5531 | 0.7109 |
| g__Pseudogracilibacillus | 0.005874 | 0.0108 | 0.001151 | 0.001979 | 0 | 0 | 0.004551 | 0.003447 | 0.03294 | 0.7109 |
| g__norank_f__Anaerolineaceae | 0.002171 | 0.003095 | 0.001303 | 0.002321 | 0.002208 | 0.005408 | 0.005917 | 0.009624 | 0.6097 | 0.766 |
| g__Acetobacter | 0.0009164 | 0.002245 | 0.003638 | 0.005619 | 0.0009133 | 0.001424 | 0.00645 | 0.009084 | 0.2021 | 0.7109 |
| g__Sphingopyxis | 0.007507 | 0.01839 | 0 | 0 | 0.002186 | 0.002991 | 0.002028 | 0.001784 | 0.1271 | 0.7109 |
| g__Chelativorans | 0.003285 | 0.003529 | 0.003496 | 0.008563 | 0.001586 | 0.003886 | 0.003498 | 0.005863 | 0.4933 | 0.7109 |
| g__norank_f__norank_o__norank_c__KD4-96 | 0.001133 | 0.001818 | 0.004398 | 0.006315 | 0.005696 | 0.003592 | 0.0003862 | 0.0009461 | 0.03607 | 0.7109 |
| g__Lysobacter | 0.001375 | 0.003367 | 0.002335 | 0.002879 | 0.0008382 | 0.001301 | 0.006566 | 0.006012 | 0.06698 | 0.7109 |
| g__norank_f__Xanthomonadaceae | 0.003009 | 0.003755 | 0.0003496 | 0.0008563 | 0.003481 | 0.00711 | 0.003329 | 0.003585 | 0.3137 | 0.7109 |
| g__Candidatus_Solibacter | 0.004902 | 0.002637 | 0.0004767 | 0.001168 | 0.001751 | 0.001373 | 0.002874 | 0.001968 | 0.04622 | 0.7109 |
| g__norank_f__Gemmatimonadaceae | 0.001647 | 0.00201 | 0.005757 | 0.006924 | 0.001443 | 0.001592 | 0.0009355 | 0.002292 | 0.2522 | 0.7109 |
| g__Erysipelotrichaceae_UCG-004 | 0 | 0 | 0 | 0 | 0 | 0 | 0.009774 | 0.02394 | 0.3916 | 0.7109 |
| g__Kurthia | 0.00198 | 0.003922 | 0.0003496 | 0.0008563 | 0.0009133 | 0.001424 | 0.005682 | 0.01392 | 0.8624 | 0.8927 |
| g__Faecalibacterium | 0.0008832 | 0.002163 | 0.0007873 | 0.00122 | 0 | 0 | 0.006192 | 0.01517 | 0.5832 | 0.7364 |
| g__Oscillibacter | 0 | 0 | 0.006796 | 0.01343 | 0 | 0 | 0.003665 | 0.005001 | 0.1041 | 0.7109 |
| g__unclassified_f__Burkholderiaceae | 0 | 0 | 0 | 0 | 0.003385 | 0.008291 | 0.004824 | 0.008628 | 0.08419 | 0.7109 |
| g__Oligella | 0.00198 | 0.003922 | 0.002105 | 0.002595 | 0.004231 | 0.01036 | 0.003168 | 0.006505 | 0.8512 | 0.8913 |
| g__norank_f__A4b | 0 | 0 | 0.001398 | 0.003425 | 0.009273 | 0.02271 | 0 | 0 | 0.5531 | 0.7109 |
| g__unclassified_f__Corynebacteriaceae | 0.0004416 | 0.001082 | 0.0004008 | 0.0009817 | 0.00586 | 0.008575 | 0.003483 | 0.008532 | 0.4933 | 0.7109 |
| g__[Eubacterium]_brachy_group | 0 | 0 | 0.007728 | 0.01782 | 0 | 0 | 0.0003788 | 0.0009279 | 0.235 | 0.7109 |
| g__Candidatus_Endomicrobium | 0 | 0 | 0 | 0 | 0 | 0 | 0.00733 | 0.01796 | 0.3916 | 0.7109 |
| g__Lachnoanaerobaculum | 0.0004582 | 0.001122 | 0.005987 | 0.01466 | 0 | 0 | 0.000387 | 0.000948 | 0.7747 | 0.8418 |
| g__Parabacteroides | 0.002072 | 0.002874 | 0.005696 | 0.01175 | 0.0004416 | 0.001082 | 0.00162 | 0.002841 | 0.6817 | 0.8147 |
| g__Moheibacter | 0.005655 | 0.01157 | 0.0007873 | 0.00122 | 0.002089 | 0.004064 | 0.0004678 | 0.001146 | 0.6904 | 0.8157 |
| g__Cloacibacterium | 0.0006911 | 0.001693 | 0.003747 | 0.002186 | 0 | 0 | 0.003665 | 0.008978 | 0.01443 | 0.555 |
| g__Alcaligenes | 0.002076 | 0.003932 | 0.0003496 | 0.0008563 | 0.00527 | 0.01016 | 0.001999 | 0.002286 | 0.6199 | 0.7711 |
| g__Acidipropionibacterium | 0.0003885 | 0.0009516 | 0.0006991 | 0.001713 | 0.001241 | 0.003039 | 0.007302 | 0.0168 | 0.8668 | 0.8936 |
| g__Ruminiclostridium_5 | 0.0004086 | 0.001001 | 0.004623 | 0.01012 | 0 | 0 | 0.002443 | 0.005985 | 0.4764 | 0.7109 |
| g__Prauserella | 0.001733 | 0.003199 | 0.003035 | 0.004877 | 0.0003966 | 0.0009714 | 0.002176 | 0.003577 | 0.5627 | 0.7178 |
| g__unclassified_f__Flavobacteriaceae | 0.0004582 | 0.001122 | 0 | 0 | 0.009704 | 0.02262 | 0.0009177 | 0.002248 | 0.5163 | 0.7109 |
| g__Hyphomicrobium | 0.001325 | 0.002267 | 0.005526 | 0.01354 | 0.0008832 | 0.002163 | 0.001917 | 0.002258 | 0.7558 | 0.8418 |
| g__Prevotella_2 | 0 | 0 | 0.006908 | 0.01306 | 0.0004136 | 0.001013 | 0 | 0 | 0.05929 | 0.7109 |
| g__Rikenellaceae_RC9_gut_group | 0.0004582 | 0.001122 | 0.005696 | 0.01175 | 0 | 0 | 0.001161 | 0.002844 | 0.4764 | 0.7109 |
| g__norank_f__Ilumatobacteraceae | 0.001661 | 0.00202 | 0.004314 | 0.003522 | 0.002165 | 0.001907 | 0.0024 | 0.003596 | 0.2817 | 0.7109 |
| g__norank_f__norank_o__Elsterales | 0.0046 | 0.00427 | 0.0004008 | 0.0009817 | 0.0009334 | 0.00146 | 0.002458 | 0.00207 | 0.03746 | 0.7109 |
| g__Terrimicrobium | 0 | 0 | 0.005049 | 0.0098 | 0 | 0 | 0.003177 | 0.006505 | 0.0927 | 0.7109 |
| g__Ruminiclostridium_1 | 0 | 0 | 0.001233 | 0.002001 | 0 | 0 | 0.006819 | 0.0167 | 0.2871 | 0.7109 |
| g__Erysipelothrix | 0 | 0 | 0.0008775 | 0.001367 | 0.003965 | 0.007505 | 0.002294 | 0.00562 | 0.4661 | 0.7109 |
| g__norank_f__Eggerthellaceae | 0 | 0 | 0.00989 | 0.02196 | 0 | 0 | 0.0009177 | 0.002248 | 0.262 | 0.7109 |
| g__Methylobacterium | 0.0004582 | 0.001122 | 0.0009211 | 0.002256 | 0 | 0 | 0.007069 | 0.004267 | 0.0007882 | 0.197 |
| g__Truepera | 0.001783 | 0.003315 | 0.002535 | 0.004064 | 0.0008832 | 0.002163 | 0.002556 | 0.003789 | 0.6605 | 0.8016 |
| g__Janibacter | 0.0008998 | 0.001394 | 0.003839 | 0.004191 | 0.002339 | 0.002235 | 0.004241 | 0.004892 | 0.4708 | 0.7109 |
| g__Tannerella | 0 | 0 | 0.009671 | 0.02369 | 0 | 0 | 0.001548 | 0.003792 | 0.5531 | 0.7109 |
| g__Erysipelatoclostridium | 0.0004086 | 0.001001 | 0.008091 | 0.01867 | 0 | 0 | 0 | 0 | 0.262 | 0.7109 |
| g__Anaerocolumna | 0.0008832 | 0.002163 | 0.0004008 | 0.0009817 | 0.001751 | 0.001373 | 0.005118 | 0.006521 | 0.2051 | 0.7109 |
| g__Reyranella | 0.001285 | 0.001416 | 0.004112 | 0.008875 | 0 | 0 | 0.002231 | 0.003213 | 0.267 | 0.7109 |
| g__RB41 | 0.001671 | 0.002182 | 0.003953 | 0.003499 | 0.002422 | 0.004798 | 0.002014 | 0.002306 | 0.5345 | 0.7109 |
| g__Collinsella | 0.001375 | 0.003367 | 0.005943 | 0.01456 | 0.002286 | 0.004259 | 0.0003862 | 0.0009461 | 0.8574 | 0.8913 |
| g__Odoribacter | 0 | 0 | 0.007503 | 0.01406 | 0 | 0 | 0 | 0 | 0.0203 | 0.5729 |
| g__Luedemannella | 0.001767 | 0.002215 | 0.001926 | 0.002502 | 0.003183 | 0.003422 | 0.0003862 | 0.0009461 | 0.2632 | 0.7109 |
| g__unclassified_f__Bacillaceae | 0.005923 | 0.0121 | 0.0009535 | 0.002336 | 0 | 0 | 0.0003788 | 0.0009279 | 0.4764 | 0.7109 |
| g__Marmoricola | 0.0007312 | 0.001135 | 0.006471 | 0.01333 | 0.0005198 | 0.001273 | 0.002786 | 0.004494 | 0.4992 | 0.7109 |
| g__norank_f__Dysgonomonadaceae | 0 | 0 | 0 | 0 | 0.0004136 | 0.001013 | 0.009753 | 0.02277 | 0.262 | 0.7109 |
| g__norank_f__norank_o__Acidobacteriales | 0.005522 | 0.004778 | 0.0008613 | 0.001339 | 0.0008963 | 0.001402 | 0.002789 | 0.004684 | 0.1451 | 0.7109 |
| g__norank_f__Fodinicurvataceae | 0 | 0 | 0.004926 | 0.008579 | 0.00171 | 0.002938 | 0.00124 | 0.002007 | 0.4508 | 0.7109 |
| g__Sedimentibacter | 0 | 0 | 0 | 0 | 0 | 0 | 0.006923 | 0.01696 | 0.3916 | 0.7109 |
| g__Dongia | 0.00118 | 0.001953 | 0.001588 | 0.001828 | 0.002357 | 0.002237 | 0.001699 | 0.001981 | 0.7628 | 0.8418 |
| g__norank_f__norank_o__Subgroup_2 | 0.00249 | 0.003096 | 0 | 0 | 0.001313 | 0.002099 | 0.001633 | 0.001924 | 0.2474 | 0.7109 |
| g__Aliicoccus | 0.001783 | 0.003215 | 0.00181 | 0.003337 | 0.0005198 | 0.001273 | 0.002395 | 0.003603 | 0.7406 | 0.8418 |
| g__norank_f__Methyloligellaceae | 0.0003857 | 0.0009446 | 0.002599 | 0.003405 | 0.002581 | 0.002212 | 0.001159 | 0.002838 | 0.2028 | 0.7109 |
| g__Bosea | 0.001229 | 0.00216 | 0.004034 | 0.008893 | 0.0005198 | 0.001273 | 0.000387 | 0.000948 | 0.839 | 0.8868 |
| g__Microlunatus | 0 | 0 | 0.003845 | 0.009419 | 0.0009334 | 0.00146 | 0.001322 | 0.002298 | 0.505 | 0.7109 |
| g__Kouleothrix | 0.002142 | 0.00337 | 0.003496 | 0.008563 | 0 | 0 | 0.0003862 | 0.0009461 | 0.5103 | 0.7109 |
| g__Acholeplasma | 0 | 0 | 0 | 0 | 0.0008551 | 0.001326 | 0.00448 | 0.01097 | 0.2871 | 0.7109 |
| g__norank_f__Caldilineaceae | 0.001833 | 0.004489 | 0.00287 | 0.005108 | 0 | 0 | 0.00124 | 0.002007 | 0.4661 | 0.7109 |
| g__norank_f__SC-I-84 | 0.00362 | 0.001334 | 0.0003496 | 0.0008563 | 0.001433 | 0.001584 | 0.000387 | 0.000948 | 0.004475 | 0.3098 |
| g__norank_f__norank_o__Microtrichales | 0.001816 | 0.003324 | 0.003129 | 0.004898 | 0 | 0 | 0.0004678 | 0.001146 | 0.4124 | 0.7109 |
| g__Amaricoccus | 0.0004582 | 0.001122 | 0.001782 | 0.00333 | 0.0004416 | 0.001082 | 0.002862 | 0.003887 | 0.3874 | 0.7109 |
| g__Pedomicrobium | 0.001641 | 0.001281 | 0.003203 | 0.003695 | 0.001319 | 0.00146 | 0 | 0 | 0.1516 | 0.7109 |
| g__Ruminococcaceae_UCG-005 | 0 | 0 | 0.006292 | 0.01541 | 0 | 0 | 0 | 0 | 0.3916 | 0.7109 |
| g__norank_f__Rhodothermaceae | 0.0004086 | 0.001001 | 0.002677 | 0.003427 | 0 | 0 | 0.001159 | 0.002838 | 0.181 | 0.7109 |
| g__norank_f__Rhizobiales_Incertae_Sedis | 0 | 0 | 0.004605 | 0.01128 | 0.0004997 | 0.001224 | 0.0007741 | 0.001896 | 0.7747 | 0.8418 |
| g__SN8 | 0 | 0 | 0 | 0 | 0.0004136 | 0.001013 | 0.005673 | 0.01281 | 0.262 | 0.7109 |
| g__unclassified_f__Dermacoccaceae | 0.0004086 | 0.001001 | 0.001202 | 0.002945 | 0.0003966 | 0.0009714 | 0.003096 | 0.007584 | 0.9979 | 0.9979 |
| g__Anaerostipes | 0.000797 | 0.001235 | 0.0006991 | 0.001713 | 0.0008832 | 0.002163 | 0.002709 | 0.006636 | 0.9542 | 0.9678 |
| g__Thermomonospora | 0.001212 | 0.001345 | 0.002097 | 0.005138 | 0 | 0 | 0.001708 | 0.002664 | 0.34 | 0.7109 |
| g__Candidatus_Stoquefichus | 0 | 0 | 0.001398 | 0.003425 | 0.003533 | 0.008653 | 0 | 0 | 0.5531 | 0.7109 |
| g__Flaviflexus | 0.001275 | 0.00209 | 0 | 0 | 0.0009429 | 0.001472 | 0.002709 | 0.006636 | 0.5025 | 0.7109 |
| g__norank_f__Roseiflexaceae | 0 | 0 | 0.00315 | 0.00278 | 0.001339 | 0.001488 | 0.000387 | 0.000948 | 0.01342 | 0.555 |
| g__Anaerolinea | 0.0003456 | 0.0008465 | 0.0008632 | 0.001348 | 0.002208 | 0.005408 | 0.001282 | 0.002093 | 0.8223 | 0.8732 |
| g__norank_f__Geminicoccaceae | 0 | 0 | 0.003129 | 0.004898 | 0.0007932 | 0.001943 | 0.0007725 | 0.001892 | 0.439 | 0.7109 |
| g__unclassified_k__norank_d__Bacteria | 0 | 0 | 0.0008287 | 0.001285 | 0 | 0 | 0.004095 | 0.004723 | 0.0713 | 0.7109 |
| g__Anaerovorax | 0 | 0 | 0 | 0 | 0.001403 | 0.00228 | 0.003087 | 0.005526 | 0.2063 | 0.7109 |
| g__Peredibacter | 0.001325 | 0.003245 | 0 | 0 | 0.0003966 | 0.0009714 | 0.002475 | 0.003113 | 0.2364 | 0.7109 |
| g__Plesiomonas | 0.0008667 | 0.001346 | 0.003113 | 0.00665 | 0 | 0 | 0.002394 | 0.004648 | 0.4691 | 0.7109 |
| g__Bdellovibrio | 0 | 0 | 0.004087 | 0.007582 | 0 | 0 | 0.000854 | 0.001332 | 0.07875 | 0.7109 |
| g__LNR_A2-18 | 0 | 0 | 0 | 0 | 0 | 0 | 0.004887 | 0.01197 | 0.3916 | 0.7109 |
| g__Gaiella | 0.0008438 | 0.001314 | 0.001873 | 0.002184 | 0.002588 | 0.001585 | 0.0008548 | 0.001333 | 0.1914 | 0.7109 |
| g__Ellin6067 | 0.0008171 | 0.002002 | 0.001205 | 0.0021 | 0.002809 | 0.004654 | 0.0007733 | 0.001198 | 0.6283 | 0.7737 |
| g__unclassified_c__Actinobacteria | 0.002749 | 0.006734 | 0.001859 | 0.003385 | 0 | 0 | 0.0003862 | 0.0009461 | 0.5103 | 0.7109 |
| g__norank_f__Bacteroidales_RF16_group | 0 | 0 | 0.004894 | 0.01199 | 0 | 0 | 0 | 0 | 0.3916 | 0.7109 |
| g__Shewanella | 0.0004582 | 0.001122 | 0.00251 | 0.003167 | 0 | 0 | 0.001712 | 0.002195 | 0.1672 | 0.7109 |
| g__unclassified_f__Veillonellaceae | 0 | 0 | 0.002671 | 0.004283 | 0 | 0 | 0.001559 | 0.001209 | 0.02769 | 0.6923 |
| g__norank_f__norank_o__Mollicutes_RF39 | 0 | 0 | 0.002447 | 0.005994 | 0 | 0 | 0.003665 | 0.008978 | 0.5531 | 0.7109 |
| g__Skermanella | 0 | 0 | 0.001049 | 0.002569 | 0 | 0 | 0.003177 | 0.006505 | 0.262 | 0.7109 |
| g__norank_f__Family_XIII | 0 | 0 | 0 | 0 | 0 | 0 | 0.004072 | 0.009975 | 0.3916 | 0.7109 |
| g__Ethanoligenens | 0 | 0 | 0.002353 | 0.004811 | 0.0004997 | 0.001224 | 0.00209 | 0.002031 | 0.1046 | 0.7109 |
| g__Butyricicoccus | 0 | 0 | 0.004195 | 0.01028 | 0 | 0 | 0 | 0 | 0.3916 | 0.7109 |
| g__Proteiniphilum | 0 | 0 | 0.0003496 | 0.0008563 | 0 | 0 | 0.005294 | 0.01297 | 0.5531 | 0.7109 |
| g__norank_f__Saprospiraceae | 0.0004582 | 0.001122 | 0.001049 | 0.002569 | 0.0004416 | 0.001082 | 0.002176 | 0.003577 | 0.7778 | 0.8418 |
| g__norank_f__norank_o__SJA-15 | 0 | 0 | 0.0006991 | 0.001713 | 0.004416 | 0.01082 | 0.0003862 | 0.0009461 | 0.7747 | 0.8418 |
| g__Ruminococcus_2 | 0 | 0 | 0.003547 | 0.007572 | 0.001845 | 0.00324 | 0 | 0 | 0.2076 | 0.7109 |
| g__norank_f__NS9_marine_group | 0.0004582 | 0.001122 | 0.001748 | 0.004281 | 0.002208 | 0.005408 | 0.0003862 | 0.0009461 | 0.9979 | 0.9979 |
| g__Ulvibacter | 0.002943 | 0.004751 | 0 | 0 | 0.0008197 | 0.001271 | 0.0009355 | 0.002292 | 0.2189 | 0.7109 |
| g__Subdoligranulum | 0 | 0 | 0.0004767 | 0.001168 | 0 | 0 | 0.004401 | 0.008377 | 0.235 | 0.7109 |
| g__Garicola | 0 | 0 | 0.0009535 | 0.002336 | 0 | 0 | 0.004453 | 0.002815 | 0.00315 | 0.3098 |
| g__Pseudofulvimonas | 0 | 0 | 0.001748 | 0.004281 | 0.003533 | 0.008653 | 0 | 0 | 0.5531 | 0.7109 |
| g__Treponema | 0 | 0 | 0 | 0 | 0.0004416 | 0.001082 | 0.00448 | 0.01097 | 0.5531 | 0.7109 |
| g__Methylocystis | 0 | 0 | 0.001238 | 0.001373 | 0.003091 | 0.007572 | 0.0004678 | 0.001146 | 0.3004 | 0.7109 |
| g__Lautropia | 0 | 0 | 0.003684 | 0.009025 | 0.0004416 | 0.001082 | 0.000387 | 0.000948 | 0.7747 | 0.8418 |
| g__Prevotella_1 | 0 | 0 | 0.005593 | 0.0137 | 0 | 0 | 0 | 0 | 0.3916 | 0.7109 |
| g__Levilinea | 0 | 0 | 0 | 0 | 0 | 0 | 0.004887 | 0.01197 | 0.3916 | 0.7109 |
| g__Megamonas | 0 | 0 | 0 | 0 | 0 | 0 | 0.004644 | 0.01028 | 0.09959 | 0.7109 |
| g__unclassified_f__Micromonosporaceae | 0 | 0 | 0.003861 | 0.00577 | 0 | 0 | 0.001403 | 0.003437 | 0.08419 | 0.7109 |
| g__Microvirga | 0.0008832 | 0.002163 | 0.001822 | 0.00287 | 0.0004231 | 0.001036 | 0.001241 | 0.001371 | 0.6732 | 0.8107 |
| g__Altererythrobacter | 0.0004416 | 0.001082 | 0.001604 | 0.001841 | 0.001261 | 0.001384 | 0.0007741 | 0.001896 | 0.5507 | 0.7109 |
| g__Nitrospira | 0.0004416 | 0.001082 | 0.002797 | 0.00685 | 0.0008101 | 0.001255 | 0.001323 | 0.002298 | 0.9231 | 0.9381 |
| g__norank_f__norank_o__Rhodospirillales | 0 | 0 | 0.0003496 | 0.0008563 | 0 | 0 | 0.004887 | 0.01197 | 0.5531 | 0.7109 |
| g__Methylocaldum | 0.0008301 | 0.00129 | 0.002352 | 0.00374 | 0 | 0 | 0.0009355 | 0.002292 | 0.4341 | 0.7109 |
| g__norank_f__Blastocatellaceae | 0.0004416 | 0.001082 | 0.002023 | 0.001688 | 0.0009133 | 0.001424 | 0 | 0 | 0.07543 | 0.7109 |
| g__norank_f__norank_o__Saccharimonadales | 0 | 0 | 0.003292 | 0.004528 | 0 | 0 | 0.0007741 | 0.001896 | 0.07118 | 0.7109 |
| g__Virgibacillus | 0 | 0 | 0 | 0 | 0 | 0 | 0.002423 | 0.004887 | 0.09959 | 0.7109 |
| g__norank_f__Ktedonobacteraceae | 0 | 0 | 0 | 0 | 0.0009994 | 0.002448 | 0.002753 | 0.006744 | 0.5531 | 0.7109 |
| g__CL500-29_marine_group | 0.0004582 | 0.001122 | 0.0006991 | 0.001713 | 0 | 0 | 0.002248 | 0.003271 | 0.2093 | 0.7109 |
| g__norank_f__Christensenellaceae | 0 | 0 | 0.0003496 | 0.0008563 | 0 | 0 | 0.003551 | 0.00559 | 0.235 | 0.7109 |
| g__norank_f__Beggiatoaceae | 0.002225 | 0.004247 | 0.0008613 | 0.001339 | 0.0003966 | 0.0009714 | 0 | 0 | 0.3936 | 0.7109 |
| g__Cardiobacterium | 0 | 0 | 0.002689 | 0.005529 | 0 | 0 | 0.0004588 | 0.001124 | 0.262 | 0.7109 |
| g__Sporotomaculum | 0 | 0 | 0 | 0 | 0 | 0 | 0.003031 | 0.007423 | 0.3916 | 0.7109 |
| g__Steroidobacter | 0.0008273 | 0.001286 | 0.001912 | 0.002499 | 0 | 0 | 0.00116 | 0.00194 | 0.2777 | 0.7109 |
| g__norank_f__norank_o__PLTA13 | 0.002129 | 0.002509 | 0.0003496 | 0.0008563 | 0.0004997 | 0.001224 | 0.0008548 | 0.001333 | 0.4002 | 0.7109 |
| g__Ignavigranum | 0 | 0 | 0.0004767 | 0.001168 | 0.002962 | 0.007254 | 0.0004678 | 0.001146 | 0.7747 | 0.8418 |
| g__norank_f__Acetobacteraceae | 0.001235 | 0.002007 | 0 | 0 | 0.0009133 | 0.001424 | 0.001633 | 0.001924 | 0.3285 | 0.7109 |
| g__norank_f__Paludibacteraceae | 0 | 0 | 0 | 0 | 0 | 0 | 0.003665 | 0.008978 | 0.3916 | 0.7109 |
| g__SWB02 | 0.0008832 | 0.002163 | 0.00116 | 0.001853 | 0 | 0 | 0.0003862 | 0.0009461 | 0.5103 | 0.7109 |
| g__Georgenia | 0.0004086 | 0.001001 | 0.002113 | 0.002497 | 0.0008197 | 0.001271 | 0.000387 | 0.000948 | 0.3151 | 0.7109 |
| g__Enhydrobacter | 0.0004582 | 0.001122 | 0 | 0 | 0.0003966 | 0.0009714 | 0.001343 | 0.002309 | 0.4764 | 0.7109 |
| g__Gordonibacter | 0 | 0 | 0 | 0 | 0.001269 | 0.003109 | 0.0004588 | 0.001124 | 0.5531 | 0.7109 |
| g__unclassified_f__Microbacteriaceae | 0.0009164 | 0.002245 | 0 | 0 | 0 | 0 | 0.000387 | 0.000948 | 0.5531 | 0.7109 |
| g__Thermopolyspora | 0.0004582 | 0.001122 | 0.002225 | 0.004206 | 0.0005198 | 0.001273 | 0 | 0 | 0.4764 | 0.7109 |
| g__Gluconacetobacter | 0 | 0 | 0.0003496 | 0.0008563 | 0.001338 | 0.001479 | 0.000387 | 0.000948 | 0.1348 | 0.7109 |
| g__Blastocatella | 0 | 0 | 0.0003496 | 0.0008563 | 0 | 0 | 0.001403 | 0.003437 | 0.5531 | 0.7109 |
| g__Longilinea | 0 | 0 | 0 | 0 | 0 | 0 | 0.001629 | 0.00399 | 0.3916 | 0.7109 |
| g__Roseimarinus | 0 | 0 | 0 | 0 | 0 | 0 | 0.001548 | 0.003792 | 0.3916 | 0.7109 |
| g__Anaerotruncus | 0 | 0 | 0.001398 | 0.003425 | 0 | 0 | 0 | 0 | 0.3916 | 0.7109 |
| g__Rhodomicrobium | 0.0003857 | 0.0009446 | 0.0003865 | 0.0009467 | 0.0003966 | 0.0009714 | 0 | 0 | 0.7747 | 0.8418 |
| g__Moorella | 0.0008832 | 0.002163 | 0 | 0 | 0 | 0 | 0 | 0 | 0.3916 | 0.7109 |
| g__Calothrix_PCC-6303 | 0 | 0 | 0 | 0 | 0 | 0 | 0.0008145 | 0.001995 | 0.3916 | 0.7109 |
| g__norank_f__mle1-27 | 0 | 0 | 0.0003496 | 0.0008563 | 0.0004416 | 0.001082 | 0 | 0 | 0.5531 | 0.7109 |
| g__ZOR0006 | 0 | 0 | 0 | 0 | 0 | 0 | 0.0007741 | 0.001896 | 0.3916 | 0.7109 |
| g__unclassified_f__Peptococcaceae | 0 | 0 | 0 | 0 | 0 | 0 | 0.0007576 | 0.001856 | 0.3916 | 0.7109 |
| g__norank_f__Elsteraceae | 0 | 0 | 0.0006991 | 0.001713 | 0 | 0 | 0 | 0 | 0.3916 | 0.7109 |
| g__unclassified_f__Sporolactobacillaceae | 0.001833 | 0.004489 | 0.0004008 | 0.0009817 | 0 | 0 | 0 | 0 | 0.5531 | 0.7109 |
| g__unclassified_f__Sphingomonadaceae | 0.0004416 | 0.001082 | 0 | 0 | 0.0009164 | 0.001439 | 0.0007741 | 0.001896 | 0.549 | 0.7109 |
| g__Planifilum | 0.0008832 | 0.002163 | 0.0003496 | 0.0008563 | 0 | 0 | 0.0004678 | 0.001146 | 0.7747 | 0.8418 |
| g__Myxococcus | 0 | 0 | 0 | 0 | 0 | 0 | 0.001282 | 0.002093 | 0.09959 | 0.7109 |
| g__norank_f__norank_o__norank_c__Subgroup_25 | 0 | 0 | 0.0008101 | 0.001273 | 0.0004231 | 0.001036 | 0 | 0 | 0.262 | 0.7109 |
| g__Leptolinea | 0 | 0 | 0 | 0 | 0 | 0 | 0.001201 | 0.00203 | 0.09959 | 0.7109 |
| g__Cryptanaerobacter | 0 | 0 | 0 | 0 | 0 | 0 | 0.001136 | 0.002784 | 0.3916 | 0.7109 |
| g__Curvibacter | 0 | 0 | 0.0003865 | 0.0009467 | 0 | 0 | 0.0004588 | 0.001124 | 0.5531 | 0.7109 |
| g__Phaselicystis | 0.0003857 | 0.0009446 | 0 | 0 | 0.0004416 | 0.001082 | 0 | 0 | 0.5531 | 0.7109 |
| g__Candidatus_Alysiosphaera | 0.0004582 | 0.001122 | 0.0004767 | 0.001168 | 0.0009614 | 0.001497 | 0.0003862 | 0.0009461 | 0.8225 | 0.8732 |
| g__Halomonas | 0.0008667 | 0.001346 | 0.0004767 | 0.001168 | 0.0003966 | 0.0009714 | 0.0004588 | 0.001124 | 0.8915 | 0.9135 |
| g__norank_f__norank_o__Rokubacteriales | 0 | 0 | 0.002134 | 0.004196 | 0 | 0 | 0 | 0 | 0.09959 | 0.7109 |
| g__unclassified_c__Gammaproteobacteria | 0.0004582 | 0.001122 | 0.001202 | 0.002945 | 0 | 0 | 0.0003862 | 0.0009461 | 0.7747 | 0.8418 |
| g__Vibrio | 0 | 0 | 0 | 0 | 0 | 0 | 0.001935 | 0.00474 | 0.3916 | 0.7109 |
| g__Pseudonocardia | 0.0004416 | 0.001082 | 0.0003496 | 0.0008563 | 0 | 0 | 0.0008548 | 0.001333 | 0.4764 | 0.7109 |
| g__unclassified_f__Christensenellaceae | 0 | 0 | 0.0004605 | 0.001128 | 0 | 0 | 0.0008145 | 0.001995 | 0.5531 | 0.7109 |
| g__norank_f__ML635J-40_aquatic_group | 0 | 0 | 0 | 0 | 0 | 0 | 0.001222 | 0.002993 | 0.3916 | 0.7109 |
| g__Kribbella | 0 | 0 | 0.0007361 | 0.001142 | 0.0004231 | 0.001036 | 0 | 0 | 0.2871 | 0.7109 |
| g__norank_f__TRA3-20 | 0.0003857 | 0.0009446 | 0 | 0 | 0.0005198 | 0.001273 | 0 | 0 | 0.5531 | 0.7109 |
| g__Hyphomonas | 0 | 0 | 0 | 0 | 0.0008832 | 0.002163 | 0 | 0 | 0.3916 | 0.7109 |
| g__Fonticella | 0 | 0 | 0 | 0 | 0 | 0 | 0.0007725 | 0.001892 | 0.3916 | 0.7109 |
| g__Sphaerisporangium | 0 | 0 | 0.0006991 | 0.001713 | 0 | 0 | 0 | 0 | 0.3916 | 0.7109 |
| g__unclassified_f__Atopobiaceae | 0 | 0 | 0 | 0 | 0.002115 | 0.005182 | 0.001305 | 0.002258 | 0.2871 | 0.7109 |
| g__norank_f__Cyclobacteriaceae | 0.0004086 | 0.001001 | 0 | 0 | 0.001763 | 0.002123 | 0.0008145 | 0.001995 | 0.2093 | 0.7109 |
| g__[Bacteroides]_pectinophilus_group | 0 | 0 | 0.002797 | 0.00685 | 0 | 0 | 0 | 0 | 0.3916 | 0.7109 |
| g__norank_f__Pseudonocardiaceae | 0.0003456 | 0.0008465 | 0.001748 | 0.004281 | 0 | 0 | 0.0004678 | 0.001146 | 0.7747 | 0.8418 |
| g__norank_f__Eubacteriaceae | 0 | 0 | 0.001211 | 0.001343 | 0 | 0 | 0.001273 | 0.002085 | 0.1041 | 0.7109 |
| g__Caenispirillum | 0 | 0 | 0.0003496 | 0.0008563 | 0 | 0 | 0.002036 | 0.004988 | 0.5531 | 0.7109 |
| g__Roseburia | 0.0004416 | 0.001082 | 0.002097 | 0.005138 | 0.0004136 | 0.001013 | 0 | 0 | 0.7747 | 0.8418 |
| g__GCA-900066225 | 0 | 0 | 0.002797 | 0.00685 | 0 | 0 | 0 | 0 | 0.3916 | 0.7109 |
| g__norank_f__Oligoflexaceae | 0 | 0 | 0.001382 | 0.003384 | 0 | 0 | 0.0007741 | 0.001896 | 0.5531 | 0.7109 |
| g__norank_f__Peptococcaceae | 0.0004086 | 0.001001 | 0.0006991 | 0.001713 | 0 | 0 | 0.001515 | 0.003712 | 0.7747 | 0.8418 |
| g__norank_f__MWH-CFBk5 | 0 | 0 | 0.001049 | 0.002569 | 0 | 0 | 0.0008548 | 0.001333 | 0.2871 | 0.7109 |
| g__Proteiniclasticum | 0 | 0 | 0 | 0 | 0.0008832 | 0.002163 | 0.0008145 | 0.001995 | 0.5531 | 0.7109 |
| g__Flindersiella | 0.0003885 | 0.0009516 | 0.001398 | 0.003425 | 0 | 0 | 0.000387 | 0.000948 | 0.7747 | 0.8418 |
| g__Deinococcus | 0 | 0 | 0 | 0 | 0 | 0 | 0.001548 | 0.003792 | 0.3916 | 0.7109 |
| g__Macrococcus | 0.0004582 | 0.001122 | 0.001322 | 0.002273 | 0 | 0 | 0 | 0 | 0.262 | 0.7109 |
| g__Eikenella | 0 | 0 | 0.001382 | 0.003384 | 0 | 0 | 0 | 0 | 0.3916 | 0.7109 |
| g__Vibrionimonas | 0 | 0 | 0.0008775 | 0.001367 | 0 | 0 | 0.0003862 | 0.0009461 | 0.235 | 0.7109 |
| g__Vulgatibacter | 0 | 0 | 0.00116 | 0.001853 | 0 | 0 | 0 | 0 | 0.09959 | 0.7109 |
| g__Acidisphaera | 0 | 0 | 0 | 0 | 0 | 0 | 0.001314 | 0.002294 | 0.09959 | 0.7109 |
| g__Candidatus_Berkiella | 0 | 0 | 0.0009211 | 0.002256 | 0 | 0 | 0 | 0 | 0.3916 | 0.7109 |
| g__Johnsonella | 0 | 0 | 0 | 0 | 0 | 0 | 0.001161 | 0.002844 | 0.3916 | 0.7109 |
| g__norank_f__norank_o__Babeliales | 0.0004582 | 0.001122 | 0.0004605 | 0.001128 | 0 | 0 | 0 | 0 | 0.5531 | 0.7109 |
| g__norank_f__Mitochondria | 0 | 0 | 0.0004008 | 0.0009817 | 0 | 0 | 0.0004678 | 0.001146 | 0.5531 | 0.7109 |
| g__norank_f__norank_o__norank_c__JG30-KF-CM66 | 0 | 0 | 0 | 0 | 0 | 0 | 0.001161 | 0.002844 | 0.3916 | 0.7109 |
| g__Raoultibacter | 0 | 0 | 0 | 0 | 0 | 0 | 0.0008145 | 0.001995 | 0.3916 | 0.7109 |
| g__Paraprevotella | 0 | 0 | 0 | 0 | 0 | 0 | 0.0007741 | 0.001896 | 0.3916 | 0.7109 |
| g__Eggerthella | 0.0006911 | 0.001693 | 0 | 0 | 0 | 0 | 0.0004072 | 0.0009975 | 0.5531 | 0.7109 |
| g__Clostridium_sensu_stricto_13 | 0 | 0 | 0 | 0 | 0 | 0 | 0.0007576 | 0.001856 | 0.3916 | 0.7109 |
| g__norank_f__Microscillaceae | 0 | 0 | 0.0003496 | 0.0008563 | 0 | 0 | 0.0003862 | 0.0009461 | 0.5531 | 0.7109 |
| g__Candidatus_Microthrix | 0.0006911 | 0.001693 | 0 | 0 | 0 | 0 | 0.0004072 | 0.0009975 | 0.5531 | 0.7109 |
| g__Ohtaekwangia | 0 | 0 | 0 | 0 | 0 | 0 | 0.0007741 | 0.001896 | 0.3916 | 0.7109 |
| g__norank_f__norank_o__PeM15 | 0 | 0 | 0.0006991 | 0.001713 | 0 | 0 | 0 | 0 | 0.3916 | 0.7109 |
| g__Haliangium | 0 | 0 | 0 | 0 | 0 | 0 | 0.0007725 | 0.001892 | 0.3916 | 0.7109 |
| g__Halocella | 0 | 0 | 0 | 0 | 0 | 0 | 0.0007725 | 0.001892 | 0.3916 | 0.7109 |
| g__norank_f__Holophagaceae | 0 | 0 | 0.0006991 | 0.001713 | 0 | 0 | 0 | 0 | 0.3916 | 0.7109 |
| g__Edaphobacter | 0.001746 | 0.002208 | 0.0004008 | 0.0009817 | 0.0004997 | 0.001224 | 0.0008466 | 0.001322 | 0.5524 | 0.7109 |
| g__Crossiella | 0.0008502 | 0.001319 | 0 | 0 | 0 | 0 | 0.0004588 | 0.001124 | 0.2871 | 0.7109 |
| g__Actinocatenispora | 0.0004416 | 0.001082 | 0 | 0 | 0 | 0 | 0.0004588 | 0.001124 | 0.5531 | 0.7109 |
| g__norank_f__BIrii41 | 0 | 0 | 0.0003496 | 0.0008563 | 0 | 0 | 0.0004678 | 0.001146 | 0.5531 | 0.7109 |
| g__Filifactor | 0.001766 | 0.004327 | 0 | 0 | 0 | 0 | 0.0007741 | 0.001896 | 0.5531 | 0.7109 |
| g__norank_f__bacteriap25 | 0 | 0 | 0.001546 | 0.001804 | 0.0008551 | 0.001326 | 0 | 0 | 0.08595 | 0.7109 |
| g__unclassified_c__Deltaproteobacteria | 0 | 0 | 0.001748 | 0.004281 | 0 | 0 | 0.0003862 | 0.0009461 | 0.5531 | 0.7109 |
| g__Chlamydia | 0 | 0 | 0.0004767 | 0.001168 | 0.0009429 | 0.001472 | 0.0004588 | 0.001124 | 0.5163 | 0.7109 |
| g__Sphaerobacter | 0.001037 | 0.002539 | 0.0003496 | 0.0008563 | 0 | 0 | 0.0004588 | 0.001124 | 0.7747 | 0.8418 |
| g__Taibaiella | 0 | 0 | 0 | 0 | 0.001692 | 0.004145 | 0 | 0 | 0.3916 | 0.7109 |
| g__Isosphaera | 0 | 0 | 0 | 0 | 0 | 0 | 0.001629 | 0.00399 | 0.3916 | 0.7109 |
| g__1921-2 | 0 | 0 | 0 | 0 | 0 | 0 | 0.001325 | 0.00227 | 0.09959 | 0.7109 |
| g__norank_f__JG30-KF-AS9 | 0.0007872 | 0.001233 | 0.000428 | 0.001048 | 0 | 0 | 0 | 0 | 0.262 | 0.7109 |
| g__Camelimonas | 0.0004582 | 0.001122 | 0 | 0 | 0.0003966 | 0.0009714 | 0 | 0 | 0.5531 | 0.7109 |
| g__Ureibacillus | 0 | 0 | 0.0008263 | 0.001303 | 0 | 0 | 0 | 0 | 0.09959 | 0.7109 |
| g__Harryflintia | 0 | 0 | 0.0006991 | 0.001713 | 0 | 0 | 0 | 0 | 0.3916 | 0.7109 |
| g__Campylobacter | 0 | 0 | 0.002303 | 0.00564 | 0 | 0 | 0.0007741 | 0.001896 | 0.5531 | 0.7109 |
| g__Pleomorphomonas | 0 | 0 | 0.00243 | 0.003095 | 0 | 0 | 0 | 0 | 0.0203 | 0.5729 |
| g__Leuconostoc | 0.001766 | 0.004327 | 0 | 0 | 0 | 0 | 0 | 0 | 0.3916 | 0.7109 |
| g__Streptosporangium | 0.001375 | 0.003367 | 0.0003496 | 0.0008563 | 0 | 0 | 0 | 0 | 0.5531 | 0.7109 |
| g__norank_f__Limnochordaceae | 0.0008832 | 0.002163 | 0.0008263 | 0.001303 | 0 | 0 | 0 | 0 | 0.2871 | 0.7109 |
| g__Rheinheimera | 0 | 0 | 0.0004008 | 0.0009817 | 0 | 0 | 0.001253 | 0.00138 | 0.07118 | 0.7109 |
| g__norank_f__Desulfarculaceae | 0.0003857 | 0.0009446 | 0.001242 | 0.002121 | 0 | 0 | 0 | 0 | 0.235 | 0.7109 |
| g__Thermobacillus | 0 | 0 | 0.0003496 | 0.0008563 | 0 | 0 | 0.00124 | 0.002007 | 0.235 | 0.7109 |
| g__Geobacter | 0 | 0 | 0.000428 | 0.001048 | 0.0003966 | 0.0009714 | 0 | 0 | 0.5531 | 0.7109 |
| g__Candidatus_Paracaedibacter | 0 | 0 | 0 | 0 | 0 | 0 | 0.0008145 | 0.001995 | 0.3916 | 0.7109 |
| g__Membranicola | 0 | 0 | 0.0003496 | 0.0008563 | 0 | 0 | 0.000387 | 0.000948 | 0.5531 | 0.7109 |
| g__norank_f__norank_o__norank_c__WS6_Dojkabacteria | 0 | 0 | 0.0008101 | 0.001273 | 0.002208 | 0.005408 | 0.0007935 | 0.00123 | 0.505 | 0.7109 |
| g__unclassified_f__Prevotellaceae | 0.0008171 | 0.002002 | 0.002447 | 0.005994 | 0 | 0 | 0 | 0 | 0.5531 | 0.7109 |
| g__Catabacter | 0 | 0 | 0 | 0 | 0 | 0 | 0.003258 | 0.00798 | 0.3916 | 0.7109 |
| g__Helicobacter | 0 | 0 | 0.003146 | 0.007707 | 0 | 0 | 0 | 0 | 0.3916 | 0.7109 |
| g__unclassified_f__Thermoanaerobacteraceae | 0 | 0 | 0 | 0 | 0 | 0 | 0.001894 | 0.00464 | 0.3916 | 0.7109 |
| g__Legionella | 0 | 0 | 0.001842 | 0.004512 | 0 | 0 | 0 | 0 | 0.3916 | 0.7109 |
| g__Tessaracoccus | 0.001325 | 0.003245 | 0 | 0 | 0.0004231 | 0.001036 | 0 | 0 | 0.5531 | 0.7109 |
| g__Anaerococcus | 0 | 0 | 0.0008016 | 0.001963 | 0 | 0 | 0.0009177 | 0.002248 | 0.5531 | 0.7109 |
| g__E1B-B3-114 | 0 | 0 | 0.001316 | 0.002173 | 0 | 0 | 0 | 0 | 0.09959 | 0.7109 |
| g__Megasphaera | 0 | 0 | 0.0009211 | 0.002256 | 0 | 0 | 0 | 0 | 0.3916 | 0.7109 |
| g__unclassified_f__Tannerellaceae | 0.0008832 | 0.002163 | 0 | 0 | 0 | 0 | 0 | 0 | 0.3916 | 0.7109 |
| g__Anaerolineaceae_UCG-001 | 0 | 0 | 0.0008016 | 0.001963 | 0 | 0 | 0.002851 | 0.006983 | 0.5531 | 0.7109 |
| g__unclassified_c__Bacteroidia | 0 | 0 | 0 | 0 | 0 | 0 | 0.0007741 | 0.001896 | 0.3916 | 0.7109 |
| g__Gracilibacter | 0.0008832 | 0.002163 | 0 | 0 | 0 | 0 | 0.001689 | 0.002983 | 0.262 | 0.7109 |
| g__[Eubacterium]_hallii_group | 0 | 0 | 0 | 0 | 0 | 0 | 0.002394 | 0.004648 | 0.09959 | 0.7109 |
| g__Ellin6055 | 0.0008832 | 0.002163 | 0.0006991 | 0.001713 | 0 | 0 | 0.0004678 | 0.001146 | 0.7747 | 0.8418 |
| g__Brachymonas | 0 | 0 | 0 | 0 | 0 | 0 | 0.001629 | 0.00399 | 0.3916 | 0.7109 |
| g__Sporomusa | 0 | 0 | 0 | 0 | 0 | 0 | 0.00156 | 0.001903 | 0.0203 | 0.5729 |
| g__unclassified_f__Acetobacteraceae | 0.0008301 | 0.00129 | 0 | 0 | 0 | 0 | 0.0004678 | 0.001146 | 0.2871 | 0.7109 |
| g__norank_f__PHOS-HE36 | 0 | 0 | 0.0003496 | 0.0008563 | 0.0004416 | 0.001082 | 0.0003788 | 0.0009279 | 0.7747 | 0.8418 |
| g__norank_f__KF-JG30-B3 | 0.0003885 | 0.0009516 | 0.001937 | 0.002502 | 0 | 0 | 0.0008548 | 0.001333 | 0.1906 | 0.7109 |
| g__norank_f__norank_o__norank_c__OLB14 | 0.0004086 | 0.001001 | 0.001303 | 0.002321 | 0.0004416 | 0.001082 | 0.000854 | 0.001332 | 0.8417 | 0.8879 |
| g__Bryobacter | 0.0009164 | 0.002245 | 0.000847 | 0.00132 | 0.0007932 | 0.001943 | 0 | 0 | 0.5832 | 0.7364 |
| g__Anaerofustis | 0 | 0 | 0.001398 | 0.003425 | 0.0004416 | 0.001082 | 0 | 0 | 0.5531 | 0.7109 |
| g__Candidatus_Arthromitus | 0 | 0 | 0.001748 | 0.004281 | 0 | 0 | 0 | 0 | 0.3916 | 0.7109 |
| g__Papillibacter | 0 | 0 | 0.001748 | 0.004281 | 0 | 0 | 0 | 0 | 0.3916 | 0.7109 |
| g__Prevotella_6 | 0 | 0 | 0.0009211 | 0.002256 | 0 | 0 | 0.0007741 | 0.001896 | 0.5531 | 0.7109 |
| g__Bilophila | 0 | 0 | 0 | 0 | 0 | 0 | 0.001548 | 0.003792 | 0.3916 | 0.7109 |
| g__Agathobacter | 0.0008171 | 0.002002 | 0.0008016 | 0.001963 | 0 | 0 | 0.001548 | 0.003792 | 0.7747 | 0.8418 |
| g__Rubrobacter | 0.002049 | 0.003219 | 0 | 0 | 0 | 0 | 0.0007935 | 0.00123 | 0.2022 | 0.7109 |
| g__Phenylobacterium | 0 | 0 | 0.0007504 | 0.001166 | 0.001336 | 0.001475 | 0.0004678 | 0.001146 | 0.2189 | 0.7109 |
| g__norank_f__norank_o__S085 | 0.001325 | 0.003245 | 0.0006991 | 0.001713 | 0 | 0 | 0.0003862 | 0.0009461 | 0.7747 | 0.8418 |
| g__norank_f__norank_o__RBG-13-54-9 | 0 | 0 | 0.001049 | 0.002569 | 0.0008551 | 0.001326 | 0 | 0 | 0.2871 | 0.7109 |
| g__IMCC26207 | 0.0008998 | 0.001394 | 0 | 0 | 0 | 0 | 0.0007741 | 0.001896 | 0.2871 | 0.7109 |
| g__Spirochaeta_2 | 0 | 0 | 0 | 0 | 0 | 0 | 0.001629 | 0.00399 | 0.3916 | 0.7109 |
| g__Aquabacterium | 0.001375 | 0.003367 | 0 | 0 | 0 | 0 | 0 | 0 | 0.3916 | 0.7109 |
| g__norank_f__Hydrogenedensaceae | 0 | 0 | 0 | 0 | 0 | 0 | 0.001222 | 0.002993 | 0.3916 | 0.7109 |
| g__Chroococcidiopsis_SAG_2023 | 0 | 0 | 0 | 0 | 0 | 0 | 0.001161 | 0.002844 | 0.3916 | 0.7109 |
| g__norank_f__F082 | 0 | 0 | 0 | 0 | 0 | 0 | 0.0007741 | 0.001896 | 0.3916 | 0.7109 |
| g__Rubellimicrobium | 0 | 0 | 0 | 0 | 0 | 0 | 0.0007741 | 0.001896 | 0.3916 | 0.7109 |
| g__Quinella | 0 | 0 | 0.003146 | 0.007707 | 0 | 0 | 0 | 0 | 0.3916 | 0.7109 |
| g__unclassified_o__Rhizobiales | 0 | 0 | 0.002303 | 0.00564 | 0 | 0 | 0.0007741 | 0.001896 | 0.5531 | 0.7109 |
| g__Antricoccus | 0.002959 | 0.004622 | 0 | 0 | 0 | 0 | 0 | 0 | 0.09959 | 0.7109 |
| g__Blastococcus | 0.0004416 | 0.001082 | 0.001176 | 0.00187 | 0 | 0 | 0.0007935 | 0.00123 | 0.4341 | 0.7109 |
| g__norank_f__norank_o__Subgroup_7 | 0 | 0 | 0.001961 | 0.002249 | 0.0003966 | 0.0009714 | 0 | 0 | 0.05929 | 0.7109 |
| g__Lachnotalea | 0 | 0 | 0 | 0 | 0 | 0 | 0.002298 | 0.00356 | 0.09959 | 0.7109 |
| g__Syner-01 | 0 | 0 | 0 | 0 | 0 | 0 | 0.002036 | 0.004988 | 0.3916 | 0.7109 |
| g__unclassified_o__Verrucomicrobiales | 0.0008832 | 0.002163 | 0 | 0 | 0 | 0 | 0 | 0 | 0.3916 | 0.7109 |
| g__Candidatus_Lariskella | 0 | 0 | 0.0004605 | 0.001128 | 0 | 0 | 0.000387 | 0.000948 | 0.5531 | 0.7109 |
| g__Family_XIII_AD3011_group | 0 | 0 | 0.0008016 | 0.001963 | 0 | 0 | 0 | 0 | 0.3916 | 0.7109 |
| g__Dialister | 0 | 0 | 0 | 0 | 0 | 0 | 0.003699 | 0.006004 | 0.09959 | 0.7109 |
| g__norank_f__norank_o__norank_c__BRH-c20a | 0 | 0 | 0 | 0 | 0.0004136 | 0.001013 | 0.002015 | 0.003912 | 0.262 | 0.7109 |
| g__Rhodanobacter | 0.0008171 | 0.002002 | 0.0004008 | 0.0009817 | 0.0003966 | 0.0009714 | 0.0007725 | 0.001892 | 0.9979 | 0.9979 |
| g__unclassified_f__Ktedonobacteraceae | 0 | 0 | 0 | 0 | 0.001293 | 0.002079 | 0.0008661 | 0.001345 | 0.2022 | 0.7109 |
| g__Tissierella | 0.0004582 | 0.001122 | 0 | 0 | 0.001366 | 0.002205 | 0 | 0 | 0.235 | 0.7109 |
| g__Acidibacter | 0 | 0 | 0.001271 | 0.002247 | 0 | 0 | 0.000387 | 0.000948 | 0.262 | 0.7109 |
| g__Weissella | 0 | 0 | 0.0004605 | 0.001128 | 0.0007932 | 0.001943 | 0.000387 | 0.000948 | 0.7747 | 0.8418 |
| g__norank_f__Gemmataceae | 0.001176 | 0.001301 | 0 | 0 | 0 | 0 | 0.0004588 | 0.001124 | 0.09809 | 0.7109 |
| g__DMER64 | 0 | 0 | 0 | 0 | 0 | 0 | 0.001629 | 0.00399 | 0.3916 | 0.7109 |
| g__unclassified_f__Dysgonomonadaceae | 0 | 0 | 0 | 0 | 0 | 0 | 0.001222 | 0.002993 | 0.3916 | 0.7109 |
| g__unclassified_o__Bacillales | 0 | 0 | 0 | 0 | 0 | 0 | 0.0007741 | 0.001896 | 0.3916 | 0.7109 |
| g__mle1-7 | 0 | 0 | 0.0008287 | 0.001285 | 0.0004416 | 0.001082 | 0 | 0 | 0.2871 | 0.7109 |
| g__norank_f__norank_o__Azospirillales | 0 | 0 | 0.0003496 | 0.0008563 | 0 | 0 | 0.0007733 | 0.001198 | 0.235 | 0.7109 |
